# Rhizobium Inoculation Drives the Shifting of Rhizosphere Fungal Community in a Host Genotype Dependent Manner

**DOI:** 10.3389/fmicb.2019.03135

**Published:** 2020-01-21

**Authors:** Hanyu Xu, Yongqing Yang, Yingzhe Tian, Ruineng Xu, Yongjia Zhong, Hong Liao

**Affiliations:** Root Biology Center, College of Resources and Environment, Fujian Agriculture and Forestry University, Fuzhou, China

**Keywords:** rhizobium inoculation, soybean, fungal community, rhizosphere, microbiome

## Abstract

Rhizosphere microorganisms play important roles in plant health and nutrition, and interactions among plants and microorganisms are important for establishment of root microbiomes. As yet, plant-microbe and microbe-microbe interactions in the rhizosphere remain largely mysterious. In this study, rhizosphere fungal community structure was first studied in a field experiment with two soybean cultivars contrasting in nodulation grown in two rhizobium inoculation treatments. Following this, recombinant inbred lines (RILs) contrasting in markers across three QTLs for biological nitrogen fixation (BNF) were evaluated for effects of genotype and rhizobium inoculation to the rhizosphere fungal community as assessed using ITS1 amplicon sequencing. The soybean plants tested herein not only hosted rhizosphere fungal communities that were distinct from bulk soils, but also specifically recruited and enriched *Cladosporium* from bulk soils. The resulting rhizosphere fungal communities varied among soybean genotypes, as well as, between rhizobium inoculation treatments. Besides, *Cladosporium* were mostly enriched in the rhizospheres of soybean genotypes carrying two or three favorable BNF QTLs, suggesting a close association between soybean traits associated with nodulation and those affecting the rhizosphere fungal community. This inference was bolstered by the observation that introduction of exogenous rhizobia significantly altered rhizosphere fungal communities to the point that these communities could be distinguished based on the combination of soybean genotype and whether exogenous rhizobia was applied. Interestingly, grouping of host plants by BNF QTLs also distinguished fungal community responses to rhizobium inoculation. Taken together, these results reveal that complex cross-kingdom interactions exist among host plants, symbiotic N_2_ fixing bacteria and fungal communities in the soybean rhizosphere.

## Introduction

Since colonizing land 450 million years ago, plants have co-evolved with associated microbes (Rosenberg and Zilber-Rosenberg, 2016). Roots of soil-grown plants not only function in mineral nutrient uptake but also provide sites for association with soil-inhabiting microbes in the rhizosphere (Hacquard et al., 2015). Microbes also interact with each other while establishing distinct plant microbiomes that play important roles in both the phyllosphere and rhizosphere (Agler et al., 2016). Plant microbiota include archaea, fungi, bacteria, and protists, which, on the whole, critically influence plant nutrition, health and productivity (Lindow and Brandl, 2003; Buée et al., 2009; Berendsen et al., 2012; Vorholt, 2012). Currently available research suggests that the establishment of plant root microbiomes largely depends on root exudates, and is strongly influenced by preferences for specific sets of microbial carbohydrates (Hu et al., 2018; Stringlis et al., 2018; Zhalnina et al., 2018; Huang et al., 2019). Beyond this, there is little information available on specific interactions between prokaryotic and eukaryotic microorganisms responsible for the establishment of plant-associated microbiomes (Hassani et al., 2018).

Although less abundant in soils than bacteria, fungi are nonetheless important for soil health through numerous interactions with plants and other microbes, most notably in the current context, as participants in symbiotic relationships. At present, the most documented soil fungi are arbuscular mycorrhiza fungi (AMF), which form symbiotic partnerships with over 80% of land plants (Field et al., 2015; Brundrett and Tedersoo, 2018). Current estimates associate 320,000–340,000 species of plants with more than 50,000 fungal species (van der Heijden et al., 2015). A benefit of joining mycorrhizal associations for plants is that the host plant receives mineral nutrients, especially phosphorus (P) (Bucher, 2007). Almost 70% of the total P acquired by rice enters symbiotically through AMF (Yang et al., 2012). These soil fungal networks are also more resilient than bacterial networks under drought conditions, and some might tolerate disturbances (de Vries et al., 2018; Igiehon and Babalola, 2018). Fungal and bacterial interactions affect plant participation in symbiotic relationships with mycorrhizal fungi, which further reinforces that multiple positive interkingdom interactions exist in plant-associated microbiota (Garbaye, 1994).

Soil fungal communities may also play crucial roles as biological control agents for associated plant species (Katoch and Pull, 2017; Singh et al., 2017). In agriculture, fungi are often used as biological control agents active against pests or pathogens through competition for nutrients and space, or as parasites of pests and pathogens. Entomopathogenic fungi can be used as biopesticides, as they actively kill insects. Globally, *Beauveria bassiana*, *Metarhizium* spp., *Hirsutella* spp., *Paecilomyces* (*Isaria*) spp., and *Lecanicillium lecanii* are all used as biological insecticides (Deshpande, 1999; Thomas and Read, 2007). In short, soil fungal communities acting as symbiotic partners or in tripartite interactions play a variety of crucial roles that benefit plants.

Beyond AMF contributions to plant associated communities, soil microbes also participate in many other interactions that affect plant health. For example, non-mycorrhizal species, such as *Arabidopsis thaliana*, may interact with endophytic fungi, such as *Colletotrichum tofieldiae* and *Serendipita indica*, with the result of increasing host plant acquisition of P, and thus promoting plant growth under low P conditions (Bakshi et al., 2015; Hiruma et al., 2016). Despite the ubiquity of fungal impacts on plants, some fungi are beneficial for plant and most plant-associated fungi exhibit neutral or detrimental effects on the growth of host plants (Durán et al., 2018).

Meanwhile, bacteria also participate in establishment of soil microbial communities. Recently, it has been reported that bacterial microbiota colonizing the roots of *Arabidopsis* promote plant survival by antagonizing filamentous fungal and oomycete eukaryotes (Durán et al., 2018). In the same work, they also demonstrated that the introduction of bacteria may shift fungal and oomycete communities both around roots and in the absence of host plants (Durán et al., 2018). Overall, a large variety of reports suggest that microbe-microbe interactions are essential components of plant associated communities, yet most of the complex interactions occurring within plant microbiomes remain uncharted.

As the third largest family of angiosperms, legumes not only form nitrogen (N) fixing nodules in symbiosis with rhizobia, but also establish associations with AMF to facilitate P acquisition from soils to host plant tissues (Doyle and Luckow, 2003; Herridge et al., 2008; Wang et al., 2010; Qin et al., 2011; Pandey et al., 2016). Soybean, as the most important leguminous crop in the world, provides protein and oil for growing human and livestock populations. Among the benefits resulting from associations between soybean and soil microbiota, rhizobia are known to fix large amounts of N_2_ that becomes available for soybeans and future crops, and is not replaceable in modern agricultural systems (Arslanoglu et al., 2011; Gerland et al., 2014).

Rhizobium inoculation is widely applied to promote soybean productivity and reduce N fertilization (Pandey et al., 2016). As documented in a recent report, rhizobium inoculation might also significantly influence the bacterial community in the rhizosphere of soybean plants (Zhong et al., 2019). To date, the extent to which rhizobium introduction influences fungal communities is unclear, with few interactions between rhizobium and fungal community members of soybean rhizospheres revealed. As hosts, this study included two parental soybean cultivars contrasting in nodulation (P1: low nodulation, P2: high nodulation), along with their low nodulation (LN1-4) and high nodulation (HN1-4) recombinant inbred lines (RILs) that also varied across markers for BNF (Biological Nitrogen Fixation), as determined by q^*BNF*^ QTL (Yang et al., 2017). ITS1 region amplicon sequencing was used to investigate the structure of fungal communities in bulk soils and in rhizospheres of the two parental soybean lines and their RILs grown under rhizobium inoculation and non-inoculation conditions, in order to reveal the potential complex interactions among rhizobia, soybean plants and fungal community.

## Materials and Methods

### Plant Materials and Field Trials

Two soybean cultivars contrasting in nodulation (P1, Low nodulation; P2, high nodulation), together with eight of their F_9__:__11_ recombinant inbred lines (RILs; HN1-4, four lines with high nodulation; LN1-4, four lines with low nodulation) were tested. The RIL lines were selected based on nodule number and the presence (HN) or absence (LN) of at least two QTLs responsible for biological nitrogen fixation (BNF) (Supplementary Figure S1 and Supplementary Table S8; Yang et al., 2017). Field trials were conducted from June to October in 2016 and 2017 on the experimental farm (E114.48°, N38.03°) of the Institute of Cereal and Oil crops, Shijiazhuang City, Heibei Province, China. The basic soil properties are listed in Supplementary Table S1. The rhizobia (*Bradyrhizobium* sp. Accession No.: EU825988) for the inoculation experiment were previously isolated, identified and studied in our lab, which could form symbiosis with all the tested soybean plants and showed higher nitrogenase activity than USDA110 (Chen et al., 2008). Besides, the *Bradyrhizobium* spp. strains have been used to identified the QTLs responsible for BNF (Yang et al., 2017).

All soybean accessions were cultivated in split plots containing complete blocks inoculated (R) or not inoculated (N) with rhizobia as previously described (Zhong et al., 2019). The schemes of two years’ field experimental design were shown in the Supplementary Figures S1B,C. In brief, in 2016 there are two blocks for two different rhizobium treatments. In each block, soybean parental genotypes were planted in a split plot with randomized complete blocks, in each block each parental soybean were cultivated in three plots. Totally, there were two big blocks, and 12 plots in 2016 field trial. Thirty seeds were sown in each plot of three 1.5 m rows spaced × 0.5 m apart. In the R treatment, soybean seeds were inoculated with rhizobia as previously described (Qin et al., 2011; Zhong et al., 2019). The field had been used for a wheat and soybean rotation for more than 10 years, with the most recent crop being wheat. No fertilizers were applied during soybean growth, and other field management procedures (e.g., irrigation, pest control, etc.) followed local practices. In 2017, another field trial using eight RILs was carried out in the same field, with a similar experimental design as in 2016. Totally, there were two blocks for two rhizobium treatments and two plots for each RIL in each block with randomized arrange. Hence, there were 32 plots in total in 2017.

### Sample Collection

Rhizosphere and bulk soil samples were separately collected when soybeans were at the R5 stage (Reproductive stage 5) of development according to Inceoglu et al. (2010), in which the biomass of soybean plants reach the highest. Soybean plants were harvested as whole plants by first digging out the root system and removing loose soils by shaking the roots. Rhizosphere soils were considered as that which adhered firmly to the roots and were collected using a sterile brush. Then the soil was stored in the liquid nitrogen and transported to the lab and stored at −80°C fridge till the total DNA extraction.

The 2016 parental genotype experiment included three biological replicates for each parent and each treatment, and the 2017 experiment with RILs included two biological replicates for each RIL and each treatment. Since RILs were divided into two groups and subsequently analyzed as two groups (HN or LN) according the QTLs and nodule number, hence, for each group under each treatment condition there were eight biological replicates. For each biological replicate, soils were collected from three independent soybean plants and mixed together. In total, 44 rhizosphere soil samples (12 in 2016 and 32 in 2017) and 6 bulk soil samples (3 each in 2016 and 2017) samples were collected as previously described (Zhong et al., 2019). Bulk soil samples were taken from 0 to 10 cm soils after carefully removing the surface soil from 3 sites near to soybean cultivation field.

### ITS1 Amplicon Library Preparation and Sequencing

Total soil DNA was extracted from 500 mg of soil using a PowerSoil DNA Isolation Kit (Mobio Laboratories, Carlsbad, CA, United States) according to the manufacturer’s protocol. DNA concentration and quality were determined using a NanoDrop 1000 spectrophotometer (Thermo Scientific, Waltham, United States). The ITS1 region from 1737 to 2043, which is about 300 bp length, was selected as the target sequencing region. The whole sequence of the Internal Transcribed Spacer 1 region (ITS1) amplicon sequencing libraries were generated using the TruSeq^®^ DNA PCR-Free Sample Preparation Kit (Illumina, United States) following the manufacturer’s recommendations, with index codes added using the following primers: ITS5-1737-F (5’-GGAAGTAAAAGTCGTAACAAGG-3’) and ITS2-2043-R (5’-GCTGCGTTCTTCATCGATGC-3’) (Findley et al., 2013). Library quality was monitored on the Qubit@ 2.0 Fluorometer (Thermo Scientific) and Agilent Bioanalyzer 2100 system. PCR reactions spanning the ITS1 region were carried out using the Phusion^®^ High-Fidelity PCR Master Mix with GC Buffer (New England Biolabs: NEB, United States). PCR products were purified using the QIAquick Gel Extraction Kit (Qiagen, Germany). Purified PCR products were subjected to ITS1 amplicon sequencing at the Novogene Institute using the Illumina Hiseq 2500 platform (Beijing, China).

### Sequence Analysis of the ITS1 Amplicons

Raw reads were assigned to the samples according to unique barcodes located on the primers, and both primers and barcodes were removed from the paired-end reads using FLASH (Magoč and Steven, 2011) and merged using Pandaseq (Masella et al., 2012). Low quality and chimeric reads were filtered using QIIME (Quantitative Insights Into Microbial Ecology) to obtain the effective reads, which were used for the subsequent analysis (Caporaso et al., 2010; Bokulich et al., 2013). Effective sequence were then clustered with UPARSE application (version 7.0.1001)^1^ and sequences with = 97% identity were assigned to the same OTUs (Edgar, 2013). Representative sequences for each OTU were screened for further annotation. For each representative sequence, the Unite Database^2^ (Kõljalg et al., 2013) was used to annotate taxonomic information (Caporaso et al., 2010) based on the Blast algorithm as calculated in QIIME (version = 1.7.0)^3^ (Altschul et al., 1990). After that the data for each sample was normalized to the sample with least effective reads and the afterword analysis were all based on this normalized data. A phylogenetic tree was constructed based on multiple sequence alignment conducted in MUSCLE (version 3.8.31)^4^ (Edgar, 2004). Rarefaction curves of observed species and Chao1 index, which is usually used for the estimation the richness of species in an ecosystem, were analyzed in QIIME. Normalized data were subjected to beta diversity analysis. Non-metric Multidimensional scaling (NMDS) and analysis of similarities (ANOSIM) were carried out using the Vegan package (version 2.3.0, anova.cca) in R (version 3.4.3). The Vegan package in R was also employed for constrained PCoA analysis, along with permutational ANOVA for calculating *P* values, with the number of permutations set at 999 (Oksanen et al., 2015; R Core Team, 2015). STAMP (version 2.1.3) was used to analyze significant differences in fungal taxa observed between genotypes and rhizobia inoculation treatments in Benjamini-Hochberg FDR corrected two-sided Welch’s *t*-tests (Parks et al., 2014). Online linear discriminant analysis effect size (LEfSe) analysis was performed to reveal biomarkers for the different sample groups based on a normalized OTU table^5^. For LEfSe analysis, the Kruskal–Wallis rank sum test was employed to distinguish significantly different species within groups at an alpha value of 0.05 and a threshold of 3.5.

Fungal co-occurrence networks in the soybean rhizosphere were constructed based on Spearman correlation matrices using the R package psych (version: 1.8.4) (Oksanen et al., 2015). Node connectivity, cumulative degree distribution and average path length were analyzed with psych (version: 1.8.4). To filter data, Spearman correlations were required to be statistically significant (*P* < 0.05) with correlation coefficients exceeding 0.7 (*r* > 0.7). Nodes of the network represented OTUs and edges connecting these nodes indicated high and significant correlations between the OTUs. Network images were generated using the interactive Gephi platform (Version, 9.2)^6^.

## Results

### Fungal Richness Was Similar Between Soybean Rhizospheres and Bulk Soils

To investigate the structure and composition of fungal communities in soybean rhizospheres, amplicon sequencing was employed, with targets selected based on the sequence of the ITS1 region. Sequencing from two years of field trials yielded 3,674,452 raw reads, of which, 3,519,397 could be combined. The average percentage of combined reads was 95.79%, with the average length being 222 bp (Supplementary Tables S2, S3). A total 3,378,293 high quality reads were obtained after removing low quality reads and chimeric reads. The average Q20 and Q30 of reads were 99.10 and 98.29%, respectively (Supplementary Tables S4, S5). Among high quality reads, 2,138,425 could be annotated. After performing OTU cluster analysis with a 97% similarity threshold for grouping sequences, the number of OTUs obtained for each sample ranged from 589 to 1374 (Supplementary Tables S6, S7).

**FIGURE 1:**
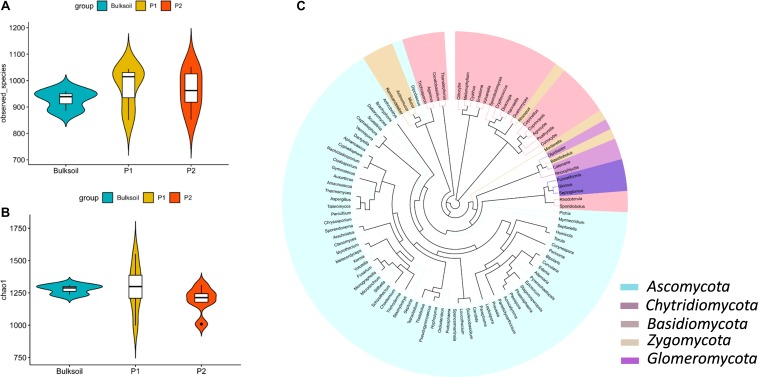
Richness and structure of soybean rhizosphere fungal communities. **(A,B)** Fungal richness was analyzed by calculating the number of observed fungal species **(A)** and Chao1 index **(B)** between bulk soil and rhizosphere soil samples collected from roots of P1 and P2 soybean plants. **(C)** Phylogenetic analysis of the top 100 soybean rhizosphere fungi in relative abundance identified at the genus level.

Rarefaction curves suggested that the sequencing depth for most of the samples was enough to discover most of the detectable fungi present in the soil over the 2 year course of these field trials (Supplementary Figure S2). The species observed and Chao1 index values indicated fungal richness was similar between bulk soil and rhizosphere samples (Figures 1A,B and Supplementary Figure S3). In contrast to this similarity between rhizospheres and bulk soil, rhizobium inoculation led to slight but not significant alterations in the richness of fungal communities (Supplementary Figure S3).

There were 831 OTUs common to both bulk soil and the rhizospheres of the two parental cultivars (Supplementary Figure S4A). For RILs, there were 880 and 906 OTUs in both bulk soil and rhizospheres of R and N soybeans, respectively (Supplementary Figures S4B,C). Other fungal OTUs specifically existed in soybean rhizospheres (Supplementary Figure S4).

Phylogenetic analysis showed that most of the high abundance fungi belonged to *Ascomycota*, *Basidiomycota*, *Chytridiomycota*, *Zygomycota*, and *Glomeromycota* at the phylum level (Figure 1C). Of these, *Ascomycota*, *Zygomycota*, and *Basidiomycota* were the dominant fungi in soybean rhizospheres, with relative abundances ranging between 45 and 65% of the total fungi counted (Supplementary Figure S5). At the family level, *Davidiellaceae*, *Cucurbitariaceae*, *Mortierellaceae*, *Rhizopodaceae*, and *Mucoraceae* were more abundant than other families of fungi (Supplementary Figures S6A,C). *Cladosporium*, *Pyrenochaetopsis*, *Rhizopus*, *Mortierella*, and *Actinomucor* were dominant at the genus taxonomic level (Supplementary Figure S6). Taken together, these results suggest that the richness of fungi in soybean rhizosphere and bulk soils mostly overlap.

### Structure and Composition Analysis of Soybean Rhizosphere Fungal Communities

**FIGURE 2:**
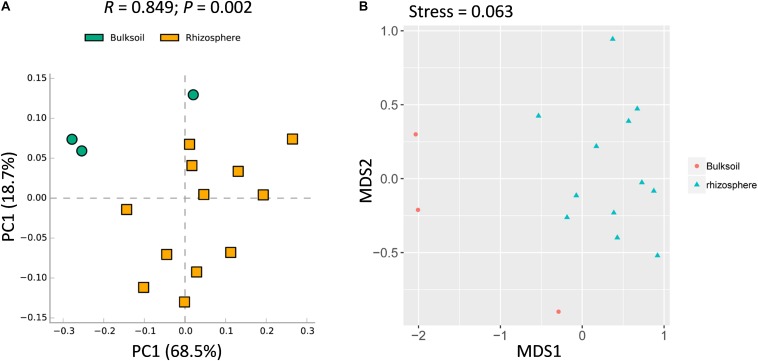
Rhizosphere fungal communities of soybean plants are distinct compared to bulk soil. **(A)** PCA analysis of bacterial communities performed using a normalized OTU and the STAMP software application (version: 2.1.3). **(B)** NMDS analysis based on Brad_Curtis distance matrix. The *R* and *P* value were calculated in the Vegan R package (version: 2.3.0) running ANOSIM analysis based on a Bray_Curtis distance matrix of bulk soil and rhizosphere samples. In the permutation test, the number of permutations tested was 999.

While overall richness of fungi did not significantly vary between rhizosphere and bulk soils, interactions within each community of microbes might still vary between soil sources. It was further analyzed at the community level. Rhizosphere soil samples were obviously shifted from bulk soil samples (Figure 2A). In addition, ANOSIM analysis suggested that the fungal communities varied significantly between bulk soil and rhizosphere samples (*R* = 0.849, *P* = 0.002) (Figure 2A). Further, similar patterns were observed with NMDS analysis, with the result that most bulk soil samples could be separated from rhizosphere samples (Stress value = 0.063) (Figure 2B). Collectively, these results demonstrate that the structure of soybean rhizosphere fungal communities is distinct from that in bulk soils, leaving open the possibility that soybean plants might drive fungal community differentiation from bulk soils to the rhizospheres.

**FIGURE 3:**
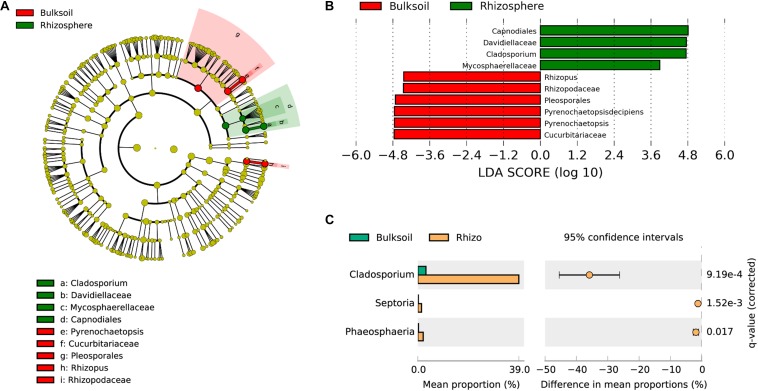
Soybean plants specifically select fungi from bulk soils. **(A)** Phylogenetic dendrogram of biomarkers identified in bulk soil and soybean rhizospheres. The circles from inside to outside indicate bacterial taxonomic levels from phylum to genus. Yellow dots represent fungi not varying significantly in relative abundance. Biomarker fungi are colored according to the corresponding class colors on the right. **(B)** LDA scores of biomarker fungi for bulk soil and rhizosphere samples. **(C)** Fungi strongly selected by soybean plants were analyzed in the STAMP software application (version: 2.1.3). Corrected *P*-values were calculated using the two-sided Welch’s *t*-test with the Benjamini–Hochberg FDR correction applied.

To analyze which fungi were significantly selected by soybean plants, LEfSe was employed to identify biomarker taxa. In these field samples, *Capnodiales*, *Davidellaceae*, *Cladosporium*, and *Mycosphaerellaceae* were identified as biomarkers for rhizosphere samples, while *Pleosporales*, *Cucurbitariaceae*, *Pyrenochaetopsis*, *Rhizopodaceae*, and *Rhizopus* were biomarkers for bulk soil samples (LDA score > 3.5; Kruskal–Wallis rank sum test, *P* < 0.05). These results suggest that soybean plants might provide resources most favorable for the proliferation of *Capnodiales*, *Davidellaceae*, and *Cladosporium* (Figures 3A,B). Further investigation of fungal families and genera favored in soybean rhizospheres as determined in the STAMP application was consistent with the results from LEfSe. *Cladosporium* were significantly enriched in soybean rhizospheres at the genus taxonomic level (adjusted *P* value < 0.001) (Figure 3C). In addition, *Septoria* and *Phaeosphaeria* were also significantly enriched by soybean plants (adjusted *P* value = 1.52e-3; *P* = 0.017). At the family level, *Davidiellaceae* (adjusted *P* = 2.81e-4), *Mycosphaerellaceae* (adjusted *P* = 1.40e-4), and *Phaeosphaeriaceae* (adjusted *P* = 0.018) were all significantly recruited by soybean plants (Supplementary Figure S7); and *Davidiellaceae* was also a biomarker in soybean rhizosphere communities (Figure 3B).

### Soybean Genotype Influences Rhizosphere Fungal Community

**FIGURE 4:**
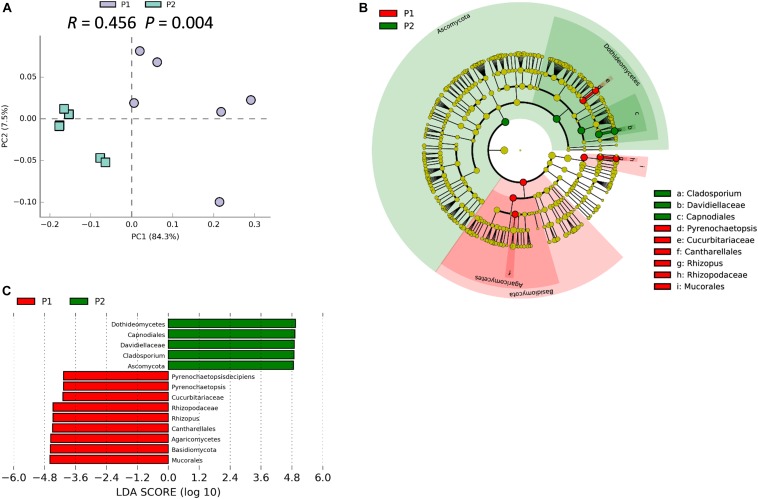
Soybean genotype has influence on rhizosphere fungal communities. **(A)** PCA of rhizosphere fungal communities associated with two parental soybean genotypes, P1 and P2, as performed based on a normalized OTU table using STAMP software. ANOSIM analysis of soybean rhizosphere fungal communities was used to calculate the difference between the two parental soybean genotypes, P1 and P2, based on the Bray_Curtis distance matrix. In the permutation test, the number of permutations was 999; *R* = 0.456, *P* = 0.004. **(B)** Biomarker fungi with LDA scores > 3.5 identified in rhizospheres surrounding roots of each parental soybean genotype as determined in the Kruskal–Wallis rank sum test, *P* < 0.05. **(C)** Phylogenetic dendrogram of biomarkers in the rhizospheres of two parental soybean genotypes. Biomarker fungi are marked according to the corresponding class colors on the right.

We previously demonstrated that host plant genotype can significantly influence the soybean rhizosphere bacterial community (Zhong et al., 2019). ANOSIM analysis indicated that the rhizosphere fungal community varied significantly between P1 and P2 samples (*R* = 0.456; *P* = 0.004), which strongly suggests that traits that vary among soybean genotypes significantly influence the rhizosphere fungal community (Figure 4A). Besides, the PCA suggested that P1 and P2 could be clearly separated by the first principal component, which explained 84.3% of variance (Figure 4A). The result of NMDS was similar with that from the PCA analysis (Stress value = 0.089) (Supplementary Figure S8). Results from LEfSe clustering of rhizosphere biomarkers at the phylum taxonomic level returned a preponderance of *Basidiomycetes* in P1 samples and *Ascomycota* in P2 samples, with P1 and P2 being clearly separated in a cladogram (Figure 4B). The identified biomarkers based on LDA scores were *Dothidemycetes*, *Capnodiales*, *Davidiellaceae*, *Cladosporium*, and *Ascomynota* for P2, along with *Mucorales*, *Basidiomycota*, *Rhizopus*, and *Pyrenochaetopsis* for P1 (Figure 4C). Taken together, these results suggest that the two parental soybean genotypes studied herein significantly influence the structure and composition of rhizosphere fungal communities in the rhizosphere.

### Rhizobium Inoculation Shifts Soybean Rhizosphere Fungal Communities

**FIGURE 5:**
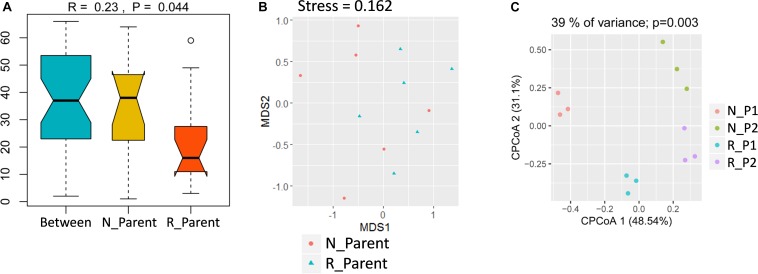
Influence of rhizobium inoculation on rhizosphere fungal communities and AMF infection rate. **(A)** Dissimilarity of fungal communities determined by ANOSIM. The *P* value was calculated by permutation testing with the number of permutation set to 999. **(B)** Differences in fungal communities of parental line rhizospheres either inoculated (R_Parent) or not inoculated (N_Parent) with rhizobium as calculated by NMDS. **(C)** Constrained principal component analysis (CPCoA) was carried out to asses clustering of rhizosphere fungal communities among N_P1, N_P2, R_P1, and R_P2 plots using a normalized OTU table and acc.anova in the Vegan R package (version: 2.3.0). The *P* value was calculated by permutation testing with the number of permutations set to 999. N_P1, Parental line1 without rhizobium inoculation; R_P1, Parental line1 with rhizobium inoculation; N_P2, Parental line 2 without rhizobium inoculation; R_P2, Parental line2 with rhizobium inoculation.

To study the impacts of rhizobium inoculation on soybean rhizosphere fungal communities, rhizosphere samples were grouped into N_Parent (Parental lines without rhizobium inoculation) and R_Parent (Parental lines with rhizobium inoculation). Analysis with ANOSIM suggested that rhizobium inoculation significantly impacted rhizosphere fungal communities (*R* = 0.24; *P* = 0.044) (Figure 5A). Further, NMDS showed that N_Parent and R_Parent rhizospheres could not be clearly separated (Figure 5B). Moreover, samples could be grouped into N_P1, N_P2, R_P1, or R_P2 categories according to the rhizobium treatment and genotype. These groups were clearly separated in CPCoA analysis, with treatment and genotype explaining 39% of the variation and significantly influencing fungal communities in the rhizosphere (*P* = 0.003) (Figure 5C). These results suggest that both soybean genotype and rhizobium inoculation exert considerable influence on the soybean rhizosphere fungal community.

**FIGURE 6:**
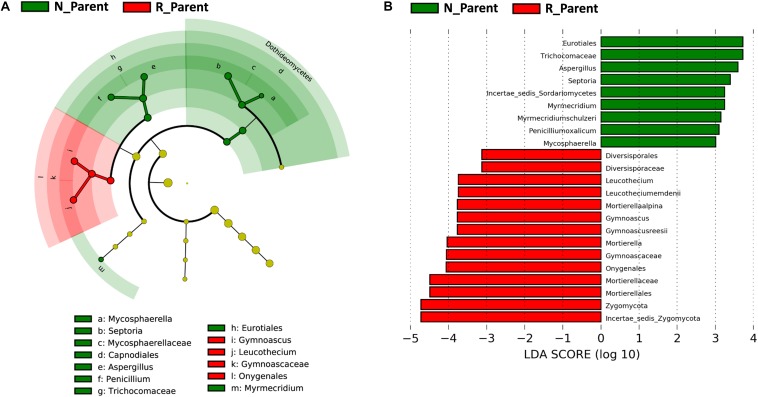
Influence of rhizobium inoculation on rhizosphere fungal communities. LEfSE analysis revealed soybean biomarkers for the rhizobium non-inoculation and inoculation treatments. **(A)** Biomarkers for the N_Parent and R_Parent rhizosphere fungi are indicated in the phylogenetic dendrogram by different colors. Red: N_Parent, Green: R_Parent. **(B)** LDA scores > 3 for biomarker fungi for N_Parent and R_Parent rhizospsheres as calculated in the Kruskal–Wallis rank sum test, *P* < 0.05. N_Parent: Parental lines grown in plots not inoculated with rhizobium; R_Parent: Parental lines grown in rhizobium inoculated plots.

Linear discriminant analysis effect size was further employed to evaluate specific fungi significantly influenced by rhizobium inoculation. In this analysis, the biomarkers for N_Parent and R_Parent were clearly separated in a cladogram (Figure 6A). *Eurotiales*, *Trichocomaceae*, *Septoria*, *Myrmecridium*, *Penicilliumoxalicum*, and *Mycosphaerella* were the biomarkers associated with R_Parent rhizospheres, while *Mortierellaalpina*, *Gymnoascus*, *Leucothecium*, and *Zygomycota* were associated with N_Parent rhizospheres (Figure 6B).

### Soybean Genotype and Rhizobium Inoculation Coordinately Influence Soybean Rhizosphere Fungal Communities

**FIGURE 7:**
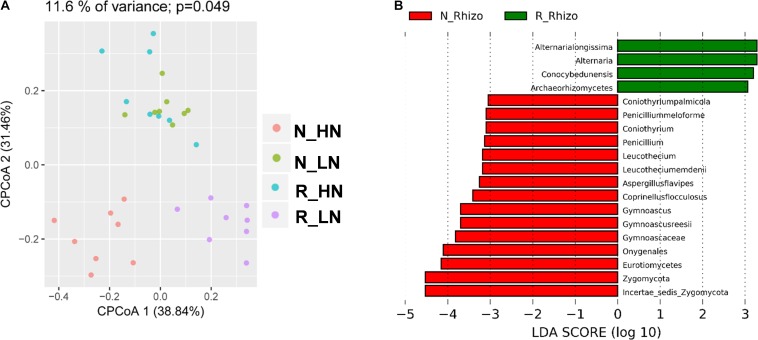
Differential responses among fungal community members to rhizobium inoculation. Data were grouped into N_HN, R_HN, N_LN, and R_LN categories to investigate the influence of nodulation traits on soybean rhizosphere fungal community responses to rhizobium inoculation. Samples were also grouped into N_Rhizo and R_Rhizo categories according to the rhizobium treatment. **(A)** CPCoA was carried out based on the Bray_Curtis distance matrix, with the *P* value calculated through ANOVA-like permutation analysis. **(B)** LDA scores > 3.0 for biomarkers of N_Rhizo and R_Rhizo grouped treatments. as calculated using the Kruskal–Wallis rank sum test, *P* < 0.05.

To investigate the extent of soybean genotypic effects on the response of fungal communities to rhizobium inoculation, we tested eight RILs selected from F_9__:__11_ recombinant inbred lines (HN1-4, four lines with high nodulation; LN1-4, four lines with low nodulation) varying in nodulation number and BNF genetic markers (Yang et al., 2017). The RILs were cultivated with (R) and without (N) rhizobium inoculation, and their rhizosphere samples were categorized into N_HN, N_LN, R_HN and R_LN groups. In CPCoA results, soybean genotype and rhizobium inoculation significantly influenced the structure of rhizosphere fungal communities (11.6% of variance; *P* = 0.049) (Figure 7A). Rhizobium inoculation significantly impacted rhizosphere fungal populations around both LN and HN roots (Figure 7B). Interestingly, R_HN rhizosphere fungal communities were not distinct from those in N_LN plots (Figure 7A). In other words, the fungal community of HN under R conditions was similar in comparison to LN under N conditions. These results suggest that rhizobium inoculation induces different responses in HN and LN soybeans.

In more granular analysis, biomarkers were compared of P1, P2, HN, and LN between different rhizobium treatment conditions, respectively. These biomarkers ended up being entirely different between tested pairs of rhizospheres, suggesting that soybean responses to rhizobium inoculation may vary among soybean genotypes (Supplementary Figures S9, S10), which was further confirmed in group comparisons of N_HN with R_HN and N_LN with R_LN (Supplementary Figures S11A,B). Taken together, our results demonstrate that soybean rhizosphere fungal community responses to rhizobium inoculation are strongly influenced by genotypic differences at a few key markers associated with N_2_ fixation.

**FIGURE 8:**
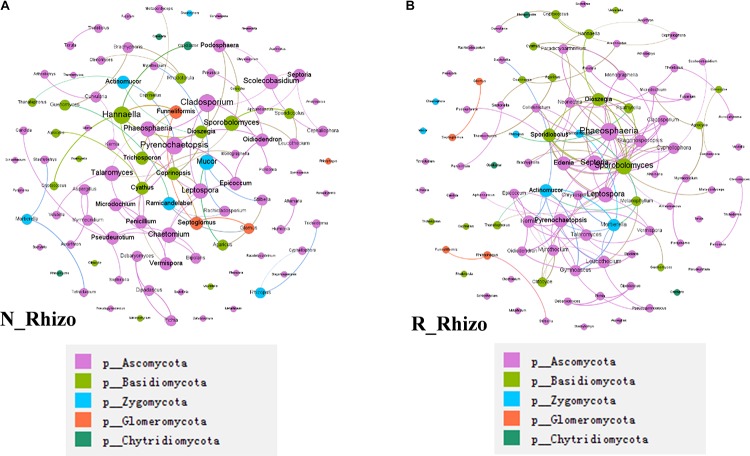
Rhizobium inoculation shifts rhizosphere fungal communities. Co-occurrence networks were constructed based on correlation analysis of the top 100 fungi in relative abundance as assessed at the genus taxonomic level. **(A)** Non-inoculated, node size is proportional to node degree, node color matches the taxa level identified for the fungus. Linkers drawn between nodes indicate significant (*P* < 0.05; Spearman’s rank correlation test) and high (Spearman’s *r* > 0.70) correlations. The number of nodes is 100 for **(A,B)**. There are 91 connections among nodes in **(A)** and 81 node connections in **(B)**. **(A)** The hub fungi for rhizospheres in non-inoculated plots were *Cladosporium*, *Pyrenochaetopsis*, *Hannaella*, *Phaeosphaeria*, and *Sporobolomyces*. **(B)** The hub fungi for LN soybean plants were *Phaeosphaeria*, *Septeria*, *Sporobolomyces*, *Leptospora*, and *Spordidobolus*. N_Rhizo: rhizosphere samples from non-inoculated plots. R_Rhizo: rhizosphere samples from rhizobium inoculated plots.

In a final analysis of impacts of rhizobium inoculation on soybean rhizosphere fungal communities, fungal co-occurrence networks were constructed for N and R communities based on the normalized OTU data. There were 100 nodes for both non-inoculated and inoculated rhizospheres. However, the number of connections varied between networks, with 78 found in the N network and 130 found in the R network (Figure 8). Plus, the hub fungi also changed with rhizobium inoculation. In non-inoculated plots, the hub rhizosphere fungi were *Cladosporium*, *Pyrenochaetopsis*, *Sporobolomyces*, *Hannaella*, and *Phaeosphaeria* (Figure 8A), while, with inoculation, the hub fungi shifted to *Phaeosphaeria*, *Sporobolomyces*, *Septoria*, *Edenia*, and *Leptospora* (Figure 8B). These results suggest that rhizobium inoculation leads to an increase in the number of connections between fungi, as well as, changes in which species act as hubs in the soybean rhizosphere fungal co-occurrence network.

## Discussion

Plant-associated microbial communities are essential for plant health and nutrient acquisition (Falkowski et al., 2008; Hacquard et al., 2015). Although microbiota have been reported in associations with many plant species (Edwards et al., 2015; Haney et al., 2015; Durán et al., 2018; Zhong et al., 2019), plant-associated fungi have received little attention. And only 5–10% of fungal community members were detectable by culture-dependent techniques (Hawksworth and Rossman, 1997; Hawksworth, 2001). In addition, interkingdom interactions among host plants and associated microbiota or among microbiota remain rarely reported. As rhizobium inoculation is a widely applied strategy in soybean production, this classic agriculture practice presents a practical means to study complex interactions between host plants and associated microbiota, including interkingdom microbial interactions between rhizobium and rhizosphere fungal community members.

### Soybean Plants Select and Enrich Some Species of Fungi in the Rhizosphere

In this study, since most OTU overlapped between rhizospheres and bulk soils, only a few specific fungi appeared to be selected in soybean rhizospheres from bulk soils (Supplementary Figure S4). This is consistent with previous reports that soil type is the major factor determining the fungal and bacterial communities, as the soil is the source of microbes available to colonize plant roots (de Ridder-Duine et al., 2005; Singh et al., 2007; Wang et al., 2009). We found no significant differences in overall fungal richness between bulk soils and rhizospheres (Figures 1A,B and Supplementary Figures S2, S3), which is also consistent with a recent report that fungal community richness only declines in the root compartment, and may not significantly vary between rhizospheres and bulk soils (Almario et al., 2017). Interestingly, this pattern stands in contrast to bacterial community differences between bulk soils and rhizospheres, wherein bacterial richness is significantly lower in the rhizosphere than in bulk soils due to selection by host plants (Bulgarelli et al., 2012; Zhong et al., 2019).

Most of soybean rhizosphere fungi detected in this study were categorized as functionally unknown. However, the most studied plant-associated fungi belonged to *Glomeromycota* and *Ectomycorrhizal* (Bonito et al., 2014). These functional known fungi might include members that benefit plants lacking associations with mycorrhizal fungi, such as *A. thaliana*, *Arabisalpina*, and *Microthlaspi* (Glynou et al., 2016; Hiruma et al., 2016; Almario et al., 2017; Hassani et al., 2018). We found that the dominant fungi in soybean rhizospheres were *Ascomycota*, *Zygomycota*, and *Basidiomycota* (Figure 1C and Supplementary Figure S5), which is consistent with previous reports that the two major phyla *Ascomycota* and *Basidiomycota* are the dominate fungi in the rhizosphere and within soybean plants (Jumpponen and Jones, 2009; Wang et al., 2009; Toju et al., 2013; Hardoim et al., 2015). Not surprisingly, *Ascomycetes* and *Basidiomycetes* have also been reported as the dominated fungi in soils (Anderson et al., 2003; Bastias et al., 2006). At the genus taxonomic level, *Cladosporium* and *Pyrenochaetopsis* were the most abundant taxa in the soybean rhizospheres observed herein (Supplementary Figure S6).

Although overall fungal community richness was similar between rhizosphere and bulk soil samples (Figure 1A and Supplementary Figure S3), PCA, NMDS, and ANOSIM analysis suggested that the structure and composition of fungal communities varied between soybean rhizospheres and bulk soils (Figure 2). This result is consistent with previous reports, in which soybean plants could recruit some species of fungi from bulk soils (Mougel et al., 2006; Wang et al., 2009; Almario et al., 2017; Zimudzi et al., 2018). In this study we further identified the fungi which were specific enriched by the soybean plant (Figures 3B,C).

Functional characterization of the fungi abundant in soybean rhizospheres is largely unexplored. Among the few known functions, a *Cladosporium* sp. isolated from soybean plants is known to exhibit GA biosynthesis activity, along with the capacity to promote plant growth (Hamayun et al., 2009). Other fungi reported to be enriched by soybean plants are *Septoria* spp. (Figure 6), which have been reported to be phytopathgens of wheat, but with unknown functions in soybean rhizospheres (Arraiano and Brown, 2017). A third type of enriched fungus, *Phaeosphaeris*, has been reported to produce a variety of chemical compounds, such as pyrazine alkaloids, isocoumarins, perylenequinones, and diterpenes (El-Demerdash, 2018). In short, though few specifics are known, soybean plants appear to select some species of fungi from bulk soils, which leads to distinctive rhizosphere fungal communities that likely fulfill specific functions in partnership with the host plants.

### Soybean Nodulation Is Associated With Rhizosphere Fungal Community Dynamics

Previous studies reported that the assembly of plant-associated fungal communities is not only dependent on soil type and plant compartments, but also on plant genotype and other environmental factors (Gottel et al., 2011; Cordier et al., 2012; Balint et al., 2013; Shakya et al., 2013). In this study, we demonstrated that soybean genotype significantly influenced the structure of rhizosphere fungal communities (Figure 4A and Supplementary Figure S8). This result stands in contrast to a former report in which no significant differences were detected among fungal communities associating with three soybean cultivars (Wang et al., 2009), possibly due to differences in profile methods, soil types and soybean genotypes used in the studies (Wang et al., 2009; Simon and Daniel, 2011; Zhong et al., 2019). This indicated the genetic traits that control rhizobium symbiosis might be also involved in the regulation of fungal community or rhizosphere fungi (Genre and Russo, 2016). Consistent with our results, a recent paper showed that the key genes in the symbiotic signaling pathway significantly influenced the enrichment of some fungi in both rhizosphere and within the roots (Thiergart et al., 2019).

In this study, *Cladosporium* sp. was significantly enriched in the rhizosphere of the high nodulation cultivar P2 (Figures 4B,C). This fungus not only was most abundant in the P2 rhizosphere, but was also exhibited higher relative abundance in high nodulation RILs compared with low nodulation RILs (Figure 3 and Supplementary Figure S6). However, *Cladosporium* was not included in a previous report on fungal communities in soybean rhizospheres (Wang et al., 2009), which might due to differences in soil types and/or soybean genotypes studied (Wieland et al., 2001; Singh et al., 2007; Wang et al., 2009). Other studies have suggested that *Cladosporium* spp. were the most frequently isolated fungi from eggplant roots (Narisawa et al., 2002) and soybean plants (Pimentel et al., 2006; Pan et al., 2008; Hamayun et al., 2009). However, outlining the exact functions of *Cladosporium* sp. in soybean microbiomes will require further experimentation. The other fungus significantly enriched in the P2 rhizosphere, *Dothideomycetes*, has been most commonly isolated from porous Antarctic rocks (Selbmann et al., 2005, 2008; Egidi et al., 2014). Whatever explanations account for selection processes in soybean rhizospheres, the results generated herein suggest that rhizosphere fungal communities are strongly influenced by variation among soybean genotypes (Supplementary Figure S9). This variation might be attributable to differences in root exudates produced by different genotypes of soybean (Ryan et al., 2001; Hu et al., 2018; Stringlis et al., 2018; Zhalnina et al., 2018; Huang et al., 2019).

### Rhizobium Inoculation Shifts the Rhizosphere Fungal Community in a Genotype Dependent Manner

Bacterial and fungal communities are the major microbial constituents of plant rhizospheres (Berendsen et al., 2012). In a previous report, we showed that rhizobium inoculation has a significant influence on the structure of rhizosphere bacterial communities in a genotype dependent manner (Zhong et al., 2019). In contrast, other studies have shown that rhizosphere fungal communities are more stable than rhizosphere bacterial communities (de Vries et al., 2018). In this study, introduction of exogenous rhizobia also changed fungal communities in soybean rhizospheres (Figure 5), with relative abundance of some fungi being significantly increased under rhizobium inoculation condition (Supplementary Figure S10A; Hamayun et al., 2009). Intriguingly, rhizobium inoculation produced different responses in the rhizospheres of two parental soybean genotypes contrasting in BNF traits and markers (Supplementary Figure S10). These results indicated that the influence of rhizobium inoculation to the fungal community is mediated by the plant genotypes, since different genotypes of soybean exhibited different responses to rhizobium inoculation. But does rhizobium inoculation influence the nitrogen status in the plant and then influenced the associated fungi needed further investigation.

After crossing the parent lines, further study with RILs varying across QTLs identified as responsible for much of BNF allowed for more in depth analysis of soybean genotypic effects on rhizosphere fungal communities in a second year of field experiments (Yang et al., 2017). The two RIL groups, categorized on the basis of BNF QTLs, varied in their rhizosphere fungal community responses to rhizobium inoculation (Figure 7A). Taken together, results from experiments with parents and RILs reinforce the conclusion that soybean rhizosphere fungal communities are impacted by soybean genotypic variation (Figure 7A and Supplementary Figures S10, S11). More specifically, QTLs identified in BNF studies, and that are known to contribute to the establishment of bacterial communities, were also found in this study to influence rhizosphere fungal communities. On the whole, results presented here suggest that soybean genotypic influences on rhizosphere fungal communities exhibit differential responses to rhizobium inoculation. While soybean rhizosphere fungal communities appear to be more stable than bacterial communities, rhizobium inoculation still influences the rhizosphere fungal community, which suggests that complex direct and indirect interactions exist between host plants and root microbiota from multiple kingdoms of organisms.

## Conclusion

In this study, we demonstrated that soybean plants could recruit certain fungi from bulk soils, which led to distinctive fungal communities in soybean rhizospheres. Soybean genotypic variation played an important role in the establishment of the rhizosphere fungal community. In addition, rhizobium inoculation also changed rhizosphere fungal communities, with the relative abundance of certain rhizosphere fungi significantly altered in directions that varied among soybean genotypes. In short, this study contributes to our understanding that complex interkingdom interactions occur in soybean rhizospheres, which might be useful in attempts to develop new strategies to improve crop performance through manipulation of rhizosphere microorganisms.

## Data Availability Statement

The raw data supporting the conclusion of this article is deposited in the NCBI: https://www.ncbi.nlm.nih.gov/bioproject/PRJNA597193.

## Author Contributions

HL, YZ, and YY designed the research. YY and RX performed the research. YZ, HX, and YT analyzed the data. YZ, HX, and HL wrote the manuscript.

## Conflict of Interest

The authors declare that the research was conducted in the absence of any commercial or financial relationships that could be construed as a potential conflict of interest.

## Footnotes

1 http://drive5.com/uparse/

2 http://unite.ut.ee/

3 http://qiime.org/scripts/assign_taxonomy.html

4 http://www.drive5.com/muscle/

5 http://huttenhower.sph.harvard.edu/galaxy/root/index

6 http://gephi.github.io/
