# Supplementary material for: Rhizobium Inoculation Drives the Shifting of Rhizosphere Fungal Community in a Host Genotype Dependent Manner
Source: Front Microbiol. 2020 Jan 21;10:3135. doi: 10.3389/fmicb.2019.03135 (PMC6985466; doi:10.3389/fmicb.2019.03135)
Supplement: Supplementary file 1 [file Presentation_1.PPTX]

## Slide 1
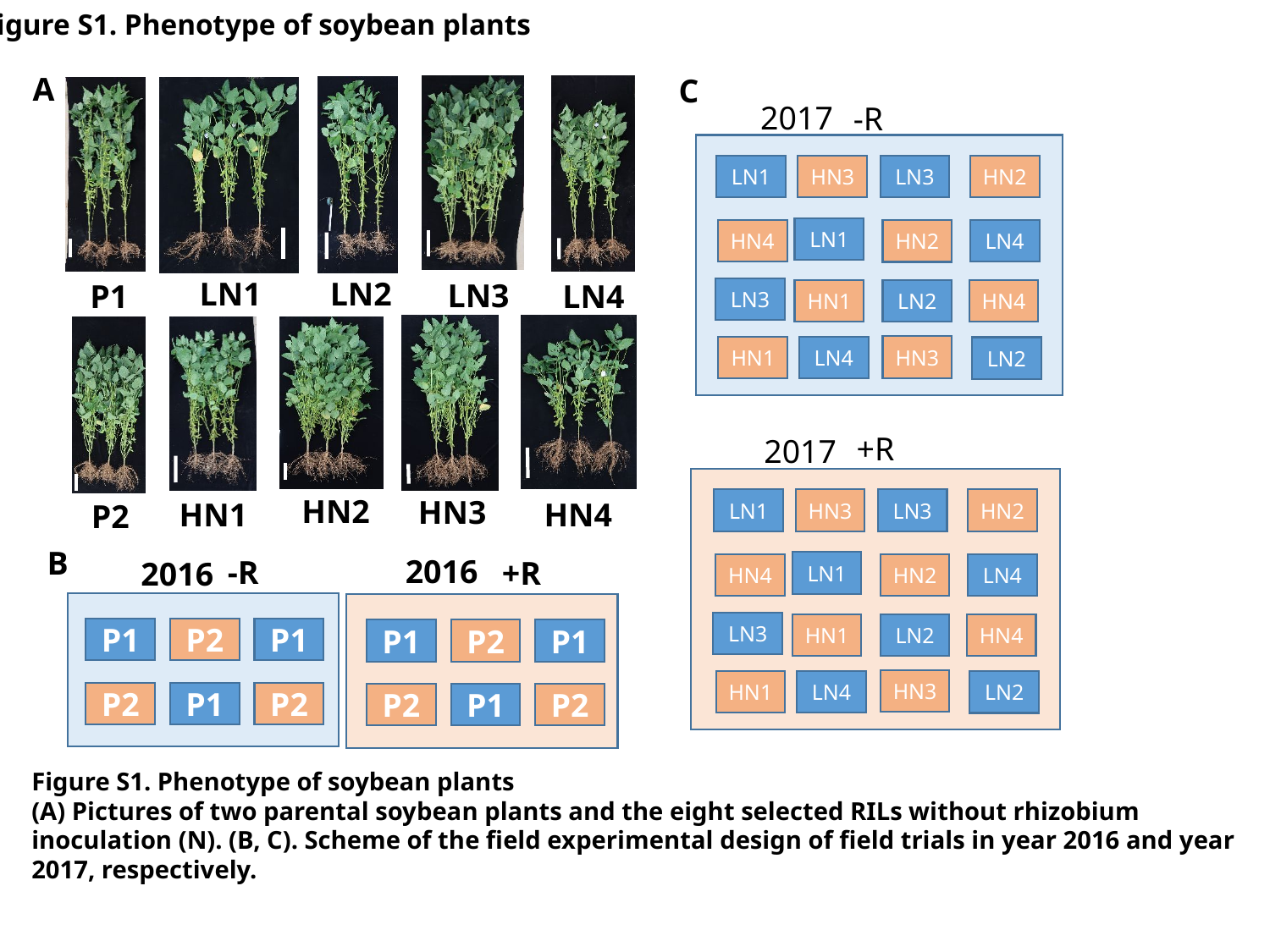

Figure S1. Phenotype of soybean plants
A
C
LN1
LN2
LN3
P1
LN4
2017
-R
HN3
LN1
LN3
HN2
LN1
HN4
LN4
HN2
LN3
HN1
LN2
HN4
HN3
LN4
HN1
LN2
+R
2017
HN3
LN1
LN3
HN2
LN1
HN4
LN4
HN2
LN3
HN1
LN2
HN4
HN3
LN4
HN1
LN2
HN2
HN3
HN4
HN1
P2
B
2016
-R
+R
2016
P2
P1
P1
P2
P1
P1
P1
P2
P2
P1
P2
P2
Figure S1. Phenotype of soybean plants
(A) Pictures of two parental soybean plants and the eight selected RILs without rhizobium inoculation (N). (B, C). Scheme of the field experimental design of field trials in year 2016 and year 2017, respectively.

## Slide 2
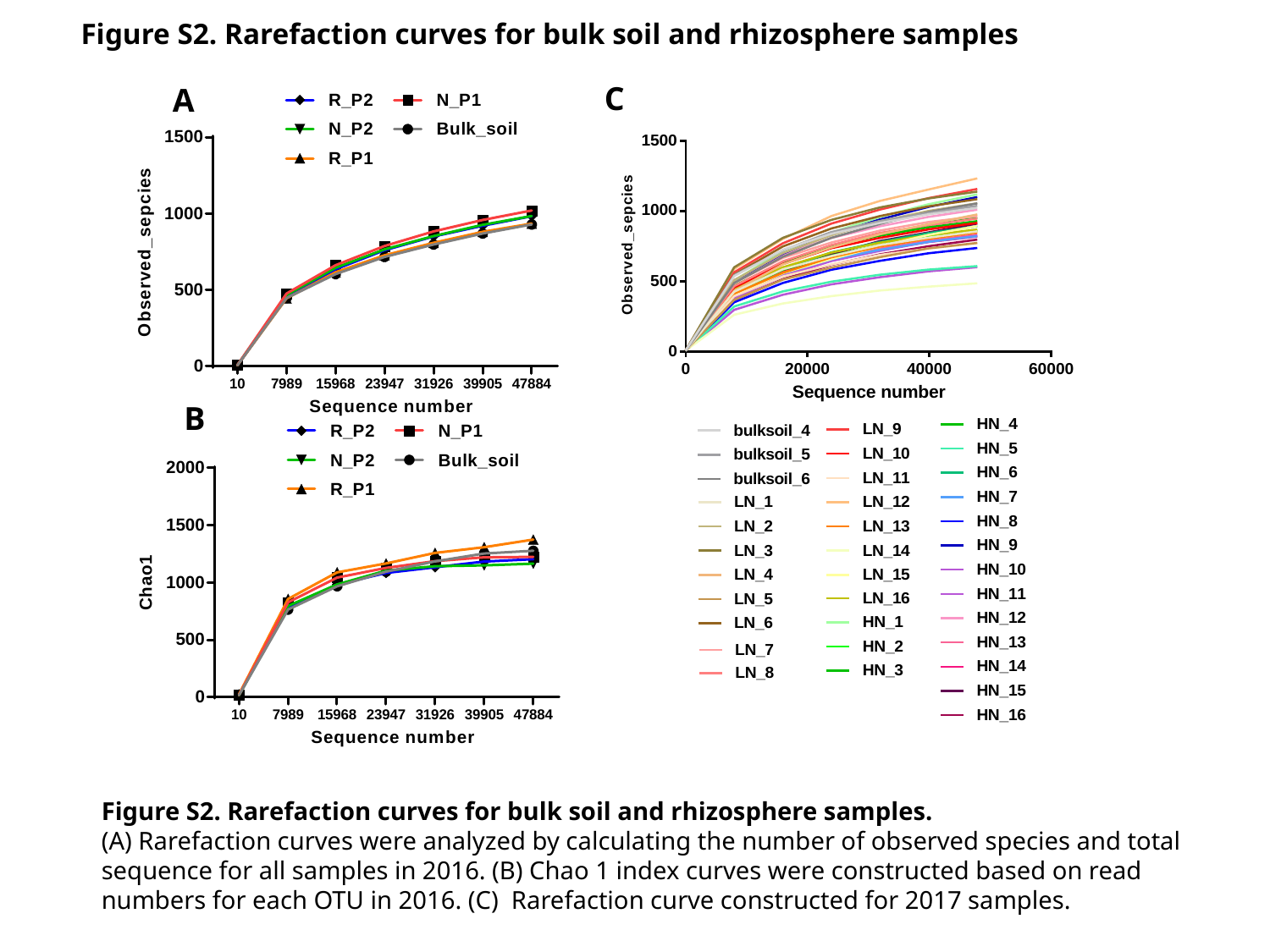

Figure S2. Rarefaction curves for bulk soil and rhizosphere samples
A
C
B
Figure S2. Rarefaction curves for bulk soil and rhizosphere samples.
(A) Rarefaction curves were analyzed by calculating the number of observed species and total sequence for all samples in 2016. (B) Chao 1 index curves were constructed based on read numbers for each OTU in 2016. (C) Rarefaction curve constructed for 2017 samples.

## Slide 3
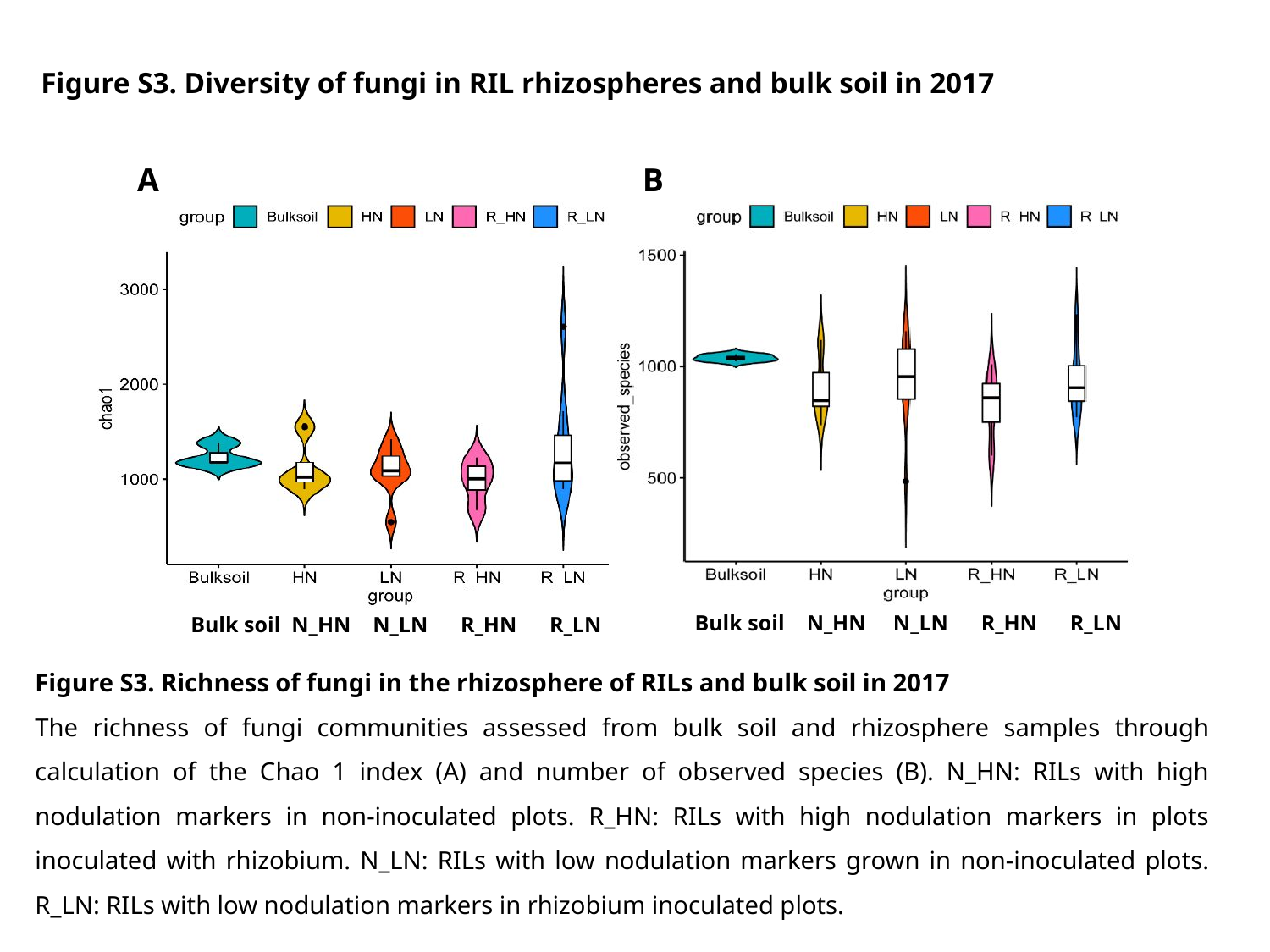

Figure S3. Diversity of fungi in RIL rhizospheres and bulk soil in 2017
A
B
Bulk soil N_HN N_LN R_HN R_LN
Bulk soil N_HN N_LN R_HN R_LN
Figure S3. Richness of fungi in the rhizosphere of RILs and bulk soil in 2017
The richness of fungi communities assessed from bulk soil and rhizosphere samples through calculation of the Chao 1 index (A) and number of observed species (B). N_HN: RILs with high nodulation markers in non-inoculated plots. R_HN: RILs with high nodulation markers in plots inoculated with rhizobium. N_LN: RILs with low nodulation markers grown in non-inoculated plots. R_LN: RILs with low nodulation markers in rhizobium inoculated plots.

## Slide 4
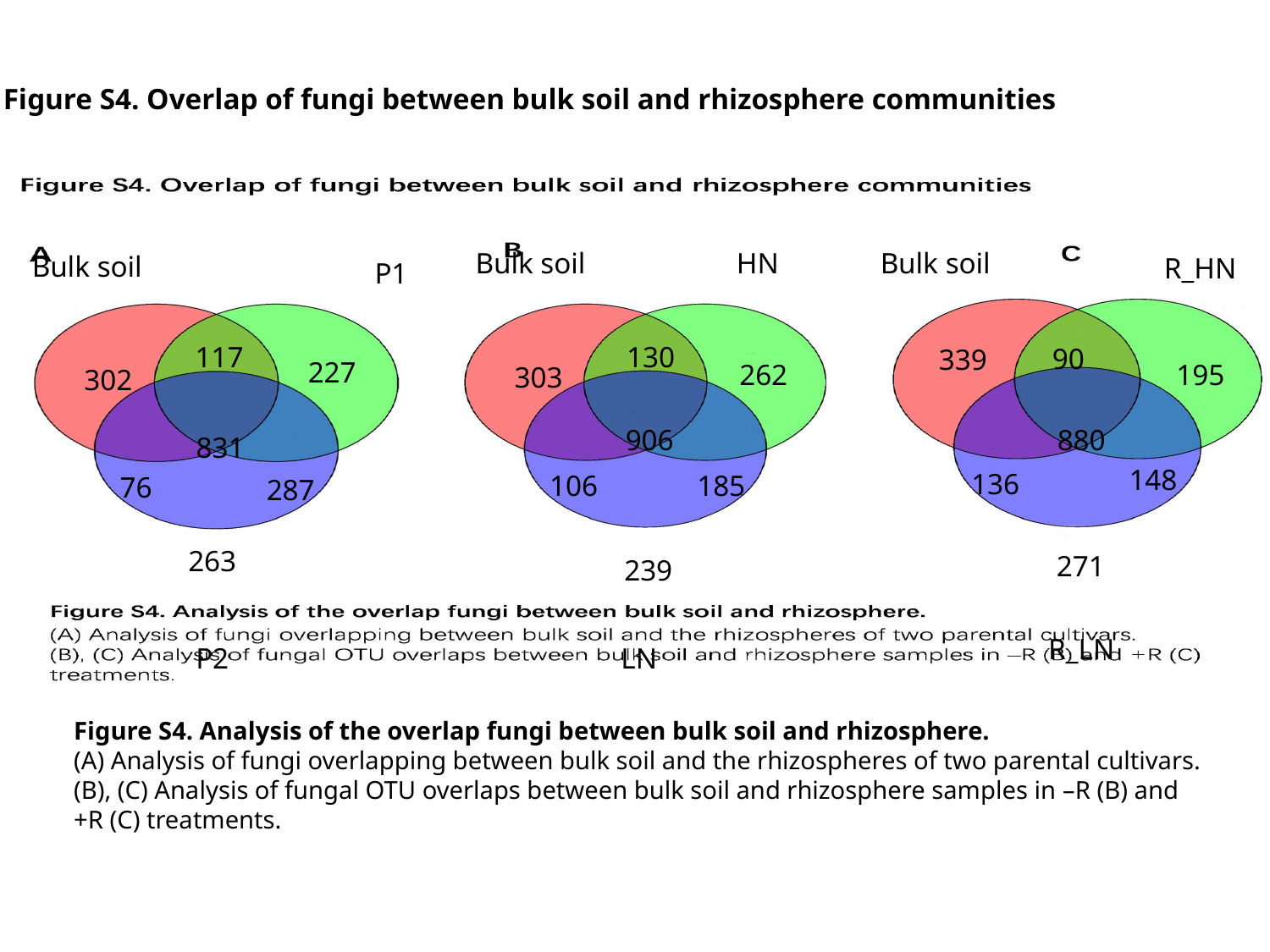

Figure S4. Overlap of fungi between bulk soil and rhizosphere communities
Bulk soil
HN
Bulk soil
Bulk soil
R_HN
P1
117
130
90
339
227
262
195
303
302
906
880
831
148
136
185
106
76
287
263
271
239
R_LN
P2
LN
Figure S4. Analysis of the overlap fungi between bulk soil and rhizosphere.
(A) Analysis of fungi overlapping between bulk soil and the rhizospheres of two parental cultivars.
(B), (C) Analysis of fungal OTU overlaps between bulk soil and rhizosphere samples in –R (B) and +R (C) treatments.

## Slide 5
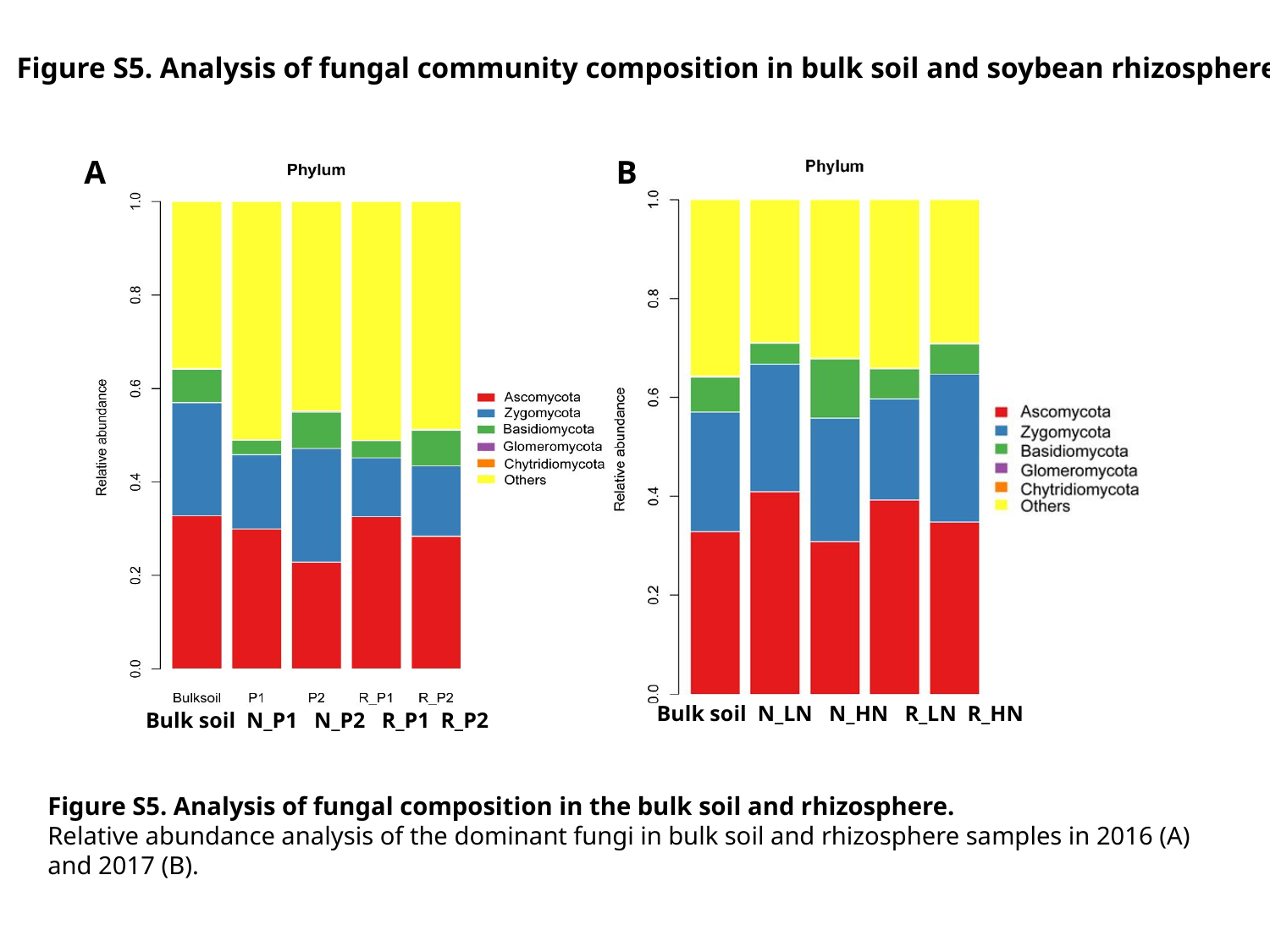

Figure S5. Analysis of fungal community composition in bulk soil and soybean rhizospheres
A
B
Bulk soil N_LN N_HN R_LN R_HN
Bulk soil N_P1 N_P2 R_P1 R_P2
Figure S5. Analysis of fungal composition in the bulk soil and rhizosphere.
Relative abundance analysis of the dominant fungi in bulk soil and rhizosphere samples in 2016 (A) and 2017 (B).

## Slide 6
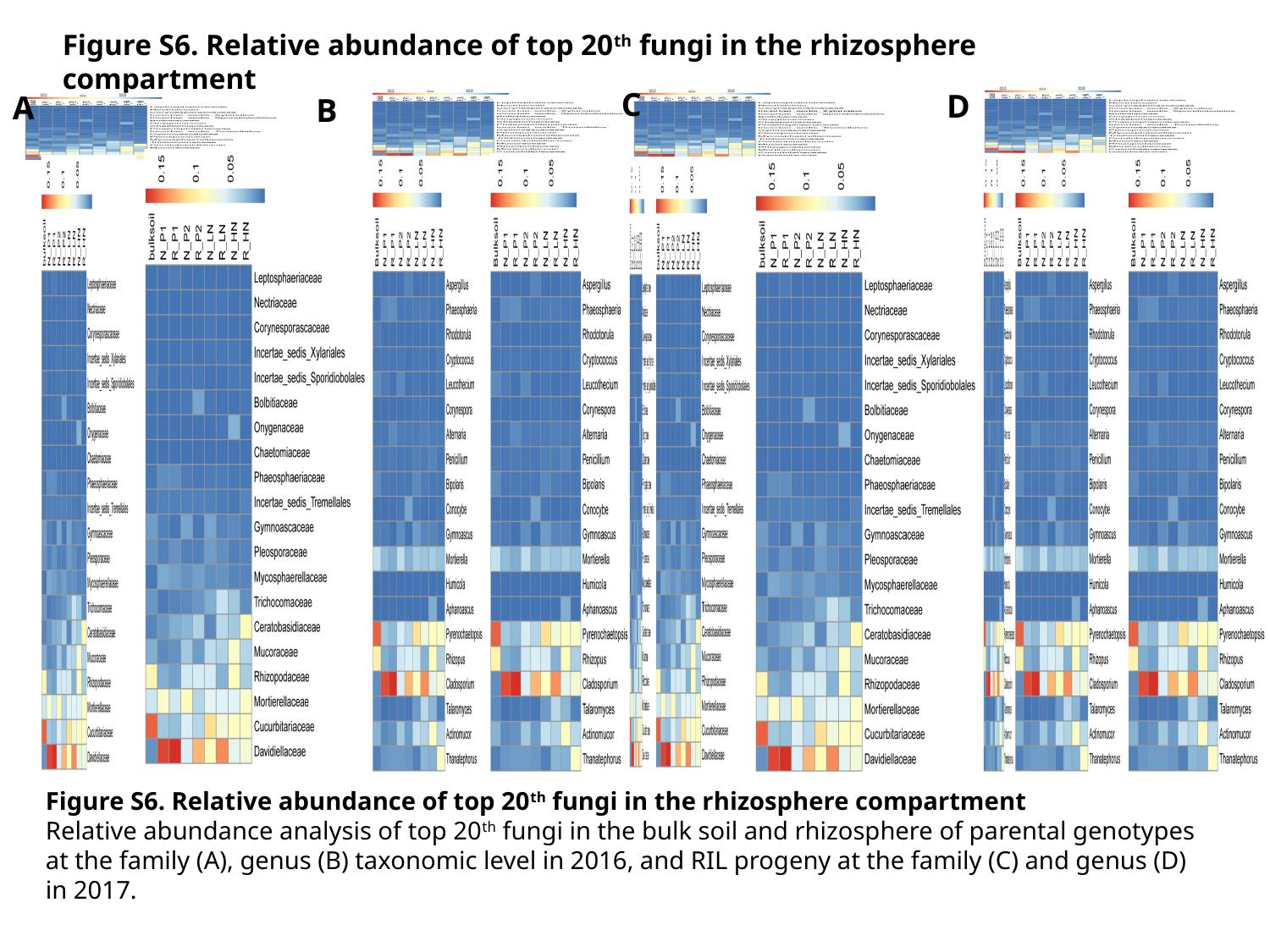

Figure S6. Relative abundance of top 20th fungi in the rhizosphere compartment
C
D
A
B
Figure S6. Relative abundance of top 20th fungi in the rhizosphere compartment
Relative abundance analysis of top 20th fungi in the bulk soil and rhizosphere of parental genotypes at the family (A), genus (B) taxonomic level in 2016, and RIL progeny at the family (C) and genus (D) in 2017.

## Slide 7
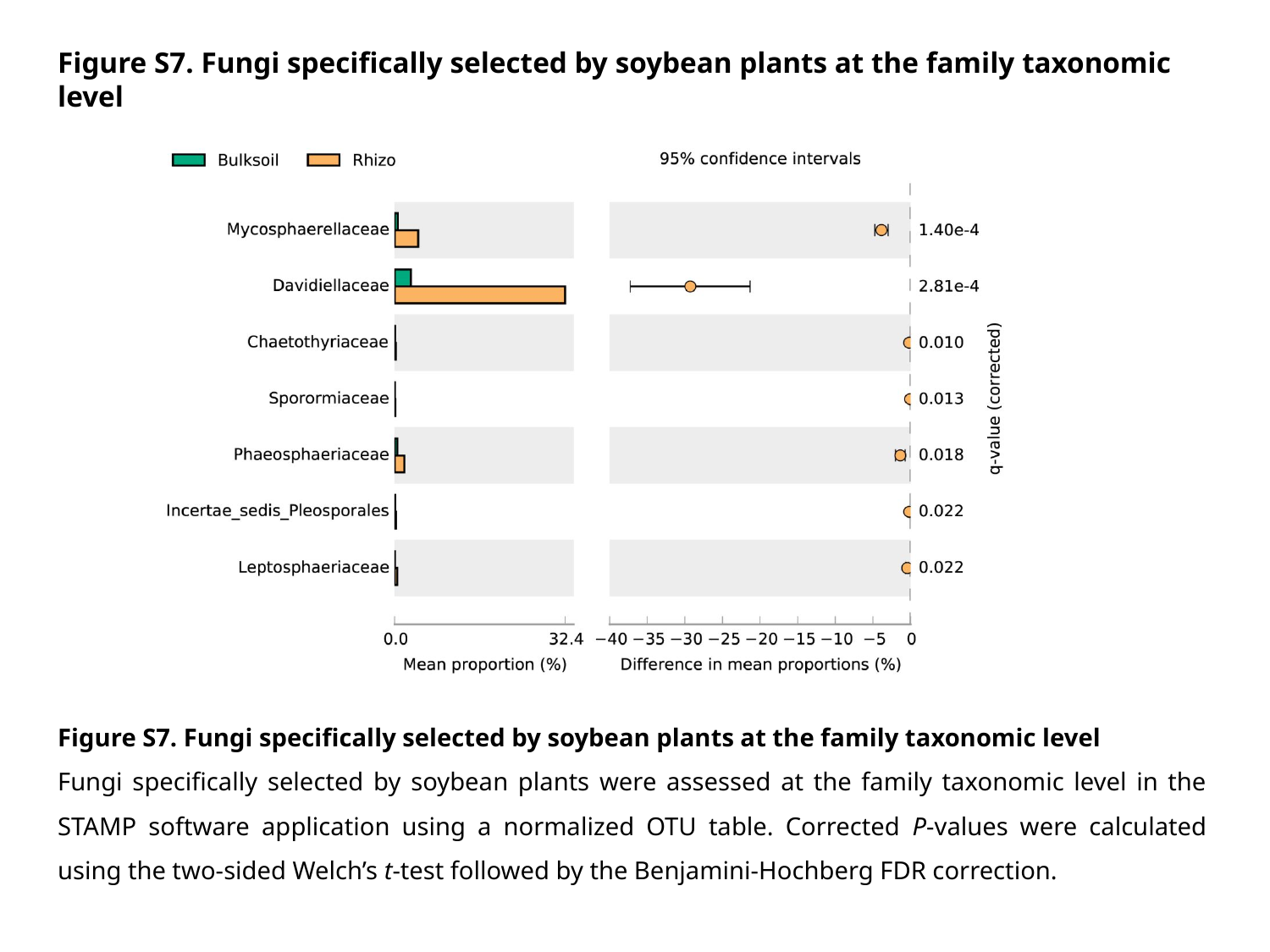

Figure S7. Fungi specifically selected by soybean plants at the family taxonomic level
Figure S7. Fungi specifically selected by soybean plants at the family taxonomic level
Fungi specifically selected by soybean plants were assessed at the family taxonomic level in the STAMP software application using a normalized OTU table. Corrected P-values were calculated using the two-sided Welch’s t-test followed by the Benjamini-Hochberg FDR correction.

## Slide 8
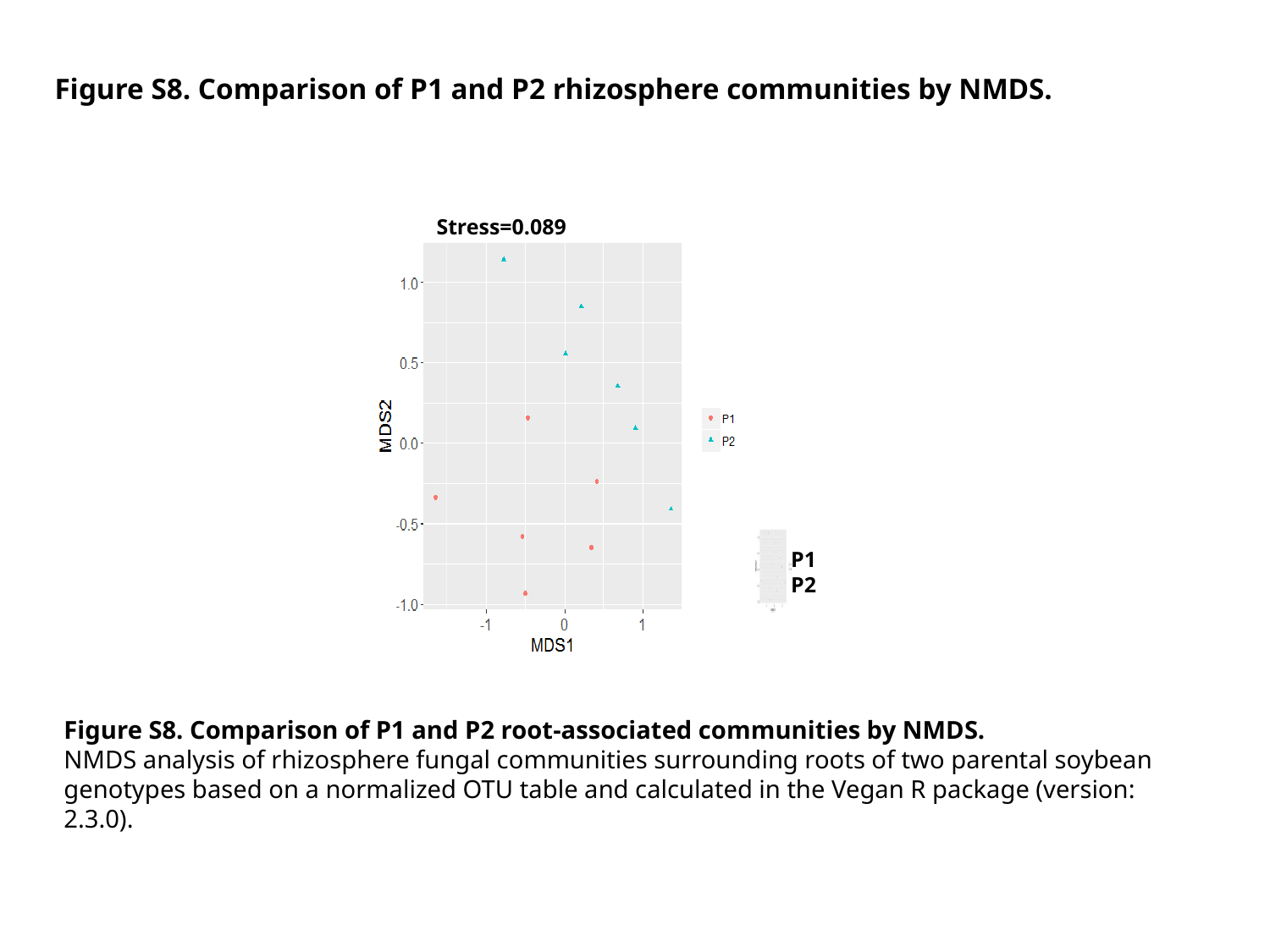

Figure S8. Comparison of P1 and P2 rhizosphere communities by NMDS.
Stress=0.089
P1
P2
Figure S8. Comparison of P1 and P2 root-associated communities by NMDS.
NMDS analysis of rhizosphere fungal communities surrounding roots of two parental soybean genotypes based on a normalized OTU table and calculated in the Vegan R package (version: 2.3.0).

## Slide 9
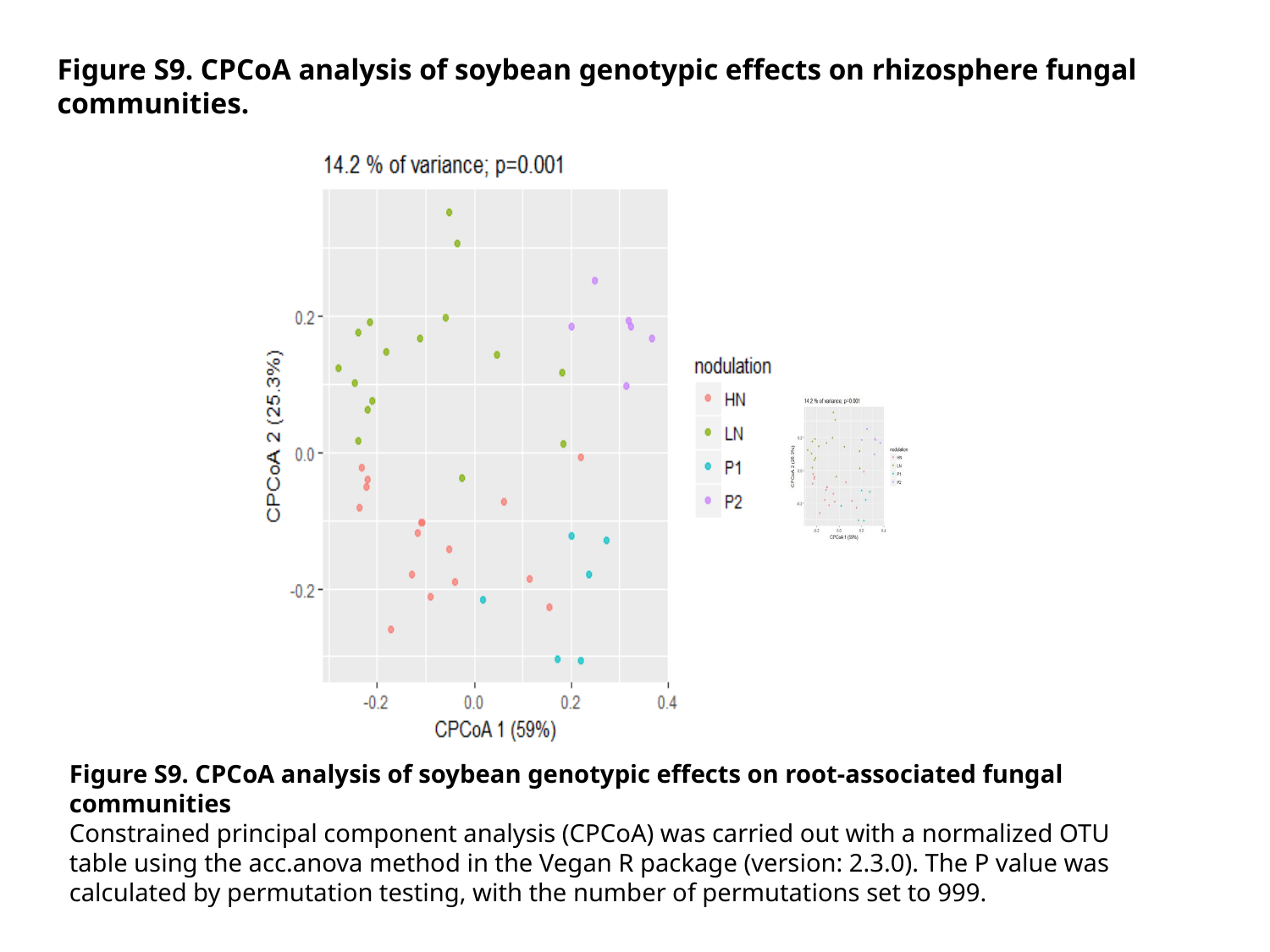

Figure S9. CPCoA analysis of soybean genotypic effects on rhizosphere fungal communities.
Figure S9. CPCoA analysis of soybean genotypic effects on root-associated fungal communities
Constrained principal component analysis (CPCoA) was carried out with a normalized OTU table using the acc.anova method in the Vegan R package (version: 2.3.0). The P value was calculated by permutation testing, with the number of permutations set to 999.

## Slide 10
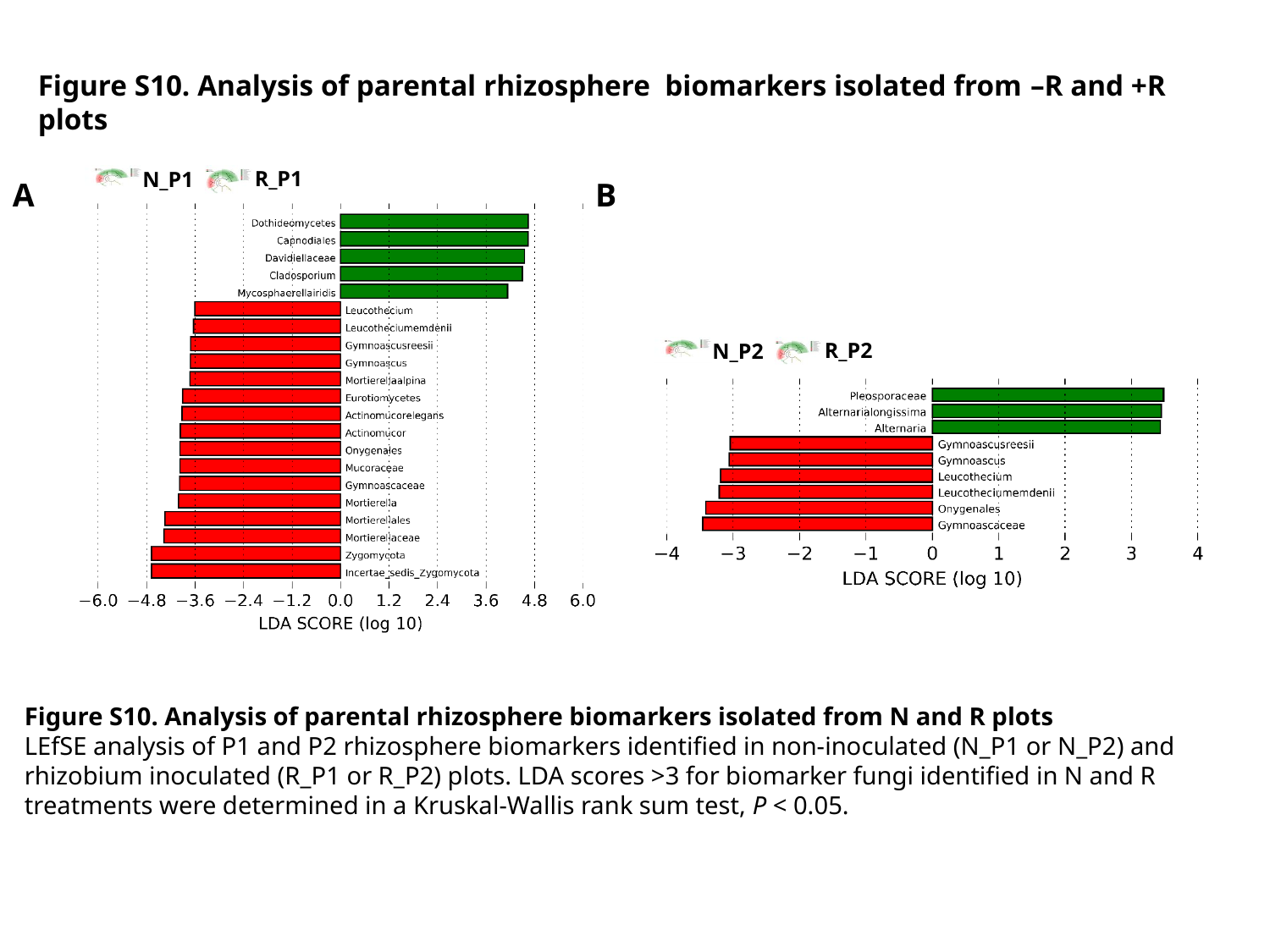

Figure S10. Analysis of parental rhizosphere biomarkers isolated from –R and +R plots
R_P1
N_P1
A
B
R_P2
N_P2
Figure S10. Analysis of parental rhizosphere biomarkers isolated from N and R plots
LEfSE analysis of P1 and P2 rhizosphere biomarkers identified in non-inoculated (N_P1 or N_P2) and rhizobium inoculated (R_P1 or R_P2) plots. LDA scores >3 for biomarker fungi identified in N and R treatments were determined in a Kruskal-Wallis rank sum test, P < 0.05.

## Slide 11
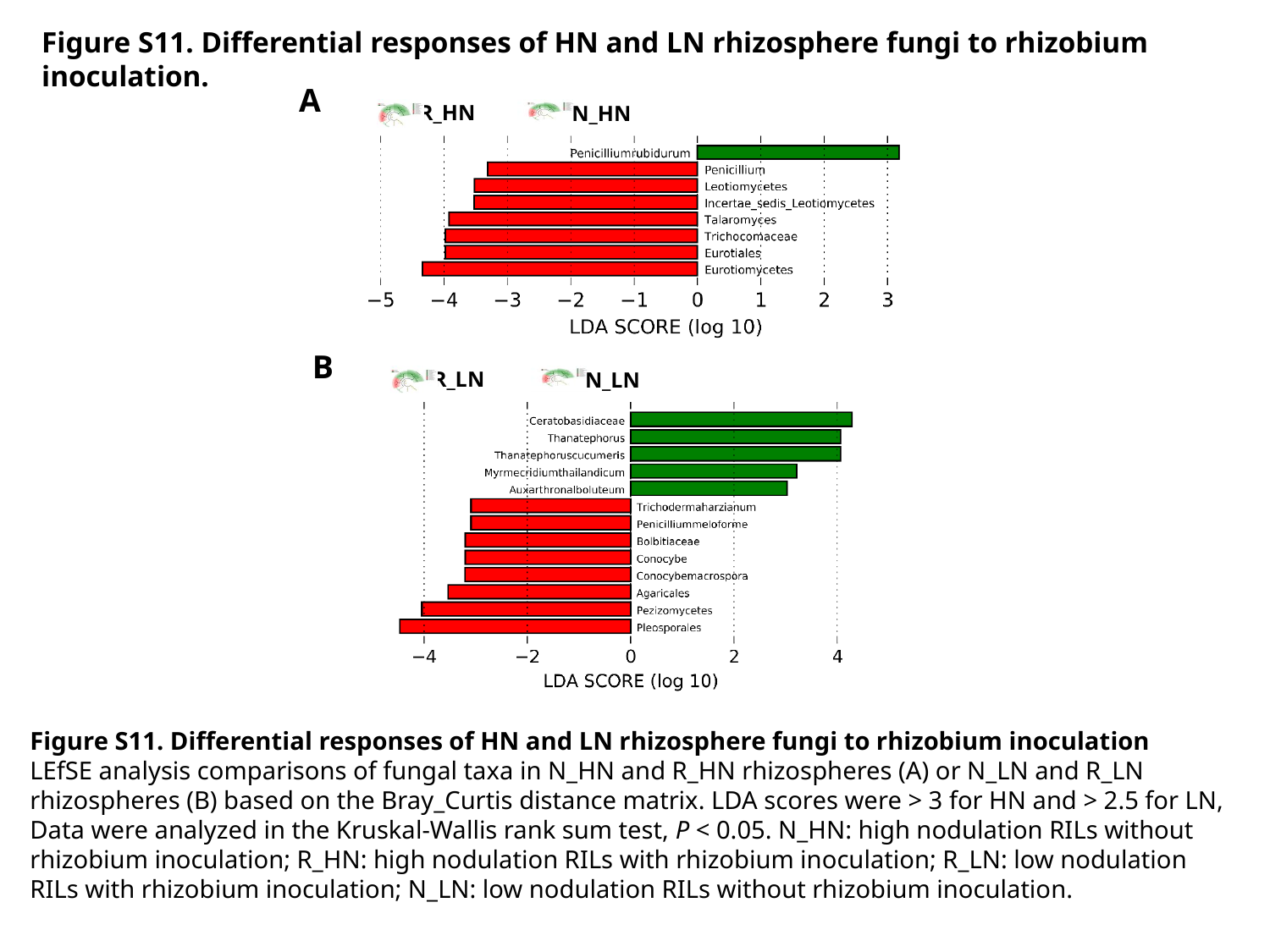

Figure S11. Differential responses of HN and LN rhizosphere fungi to rhizobium inoculation.
A
R_HN
N_HN
B
R_LN
N_LN
Figure S11. Differential responses of HN and LN rhizosphere fungi to rhizobium inoculation
LEfSE analysis comparisons of fungal taxa in N_HN and R_HN rhizospheres (A) or N_LN and R_LN rhizospheres (B) based on the Bray_Curtis distance matrix. LDA scores were > 3 for HN and > 2.5 for LN, Data were analyzed in the Kruskal-Wallis rank sum test, P < 0.05. N_HN: high nodulation RILs without rhizobium inoculation; R_HN: high nodulation RILs with rhizobium inoculation; R_LN: low nodulation RILs with rhizobium inoculation; N_LN: low nodulation RILs without rhizobium inoculation.
