# Supplementary material for: Rhizobium Inoculation Drives the Shifting of Rhizosphere Fungal Community in a Host Genotype Dependent Manner
Source: Front Microbiol. 2020 Jan 21;10:3135. doi: 10.3389/fmicb.2019.03135 (PMC6985466; doi:10.3389/fmicb.2019.03135)
Supplement: Supplementary file 2 [file Data_Sheet_1.docx]

Supplemental table S1: Basic properties of soils in the field sites

| pH | Organic matter  g.kg^-1^ | Available N  mg.kg^-1^ | Available K  mg.kg^-1^ | Available P  mg.kg^-1^ |
| --- | --- | --- | --- | --- |
| 8.53±0.13 | 20.6±2.04 | 84.7±3.19 | 159.5±6.46 | 14.5±0.34 |

Supplemental table S2: Numbers and characters of sequence reads in 2016.

| Sample name | Total reads | Combined  reads | Uncombined  reads | Combined  Percentage (%) | Combined  base(bp) | Average length (bp) |
| --- | --- | --- | --- | --- | --- | --- |
| bulksoil1 | 89,486 | 85,898 | 3,588 | 95.99 | 18,812,656 | 219 |
| bulksoil2 | 89,282 | 85,634 | 3,648 | 95.91 | 18,827,879 | 220 |
| bulksoil3 | 87,147 | 84,159 | 2,988 | 96.57 | 19,806,389 | 235 |
| N_P1_1 | 85,407 | 82,336 | 3,071 | 96.4 | 19,207,001 | 233 |
| N_P1_2 | 83,038 | 80,013 | 3,025 | 96.36 | 18,367,750 | 230 |
| N_P1_3 | 70,284 | 66,806 | 3,478 | 95.05 | 14,934,528 | 224 |
| R_P1_1 | 81,844 | 79,558 | 2,286 | 97.21 | 18,479,978 | 232 |
| R_P1_2 | 71,910 | 69,912 | 1,998 | 97.22 | 16,298,504 | 233 |
| R_P1_3 | 89,444 | 86,400 | 3,044 | 96.6 | 19,696,812 | 228 |
| N_P2_1 | 80,675 | 78,556 | 2,119 | 97.37 | 18,694,262 | 238 |
| N_P2_2 | 73,690 | 71,683 | 2,007 | 97.28 | 16,063,665 | 224 |
| N_P2_3 | 74,647 | 72,094 | 2,553 | 96.58 | 16,426,163 | 228 |
| R_P2_1 | 73,615 | 71,652 | 1,963 | 97.33 | 17,094,171 | 239 |
| R_P2_2 | 77,693 | 74,953 | 2,740 | 96.47 | 16,706,816 | 223 |
| R_P2_3 | 89,257 | 85,113 | 4,144 | 95.36 | 19,456,213 | 229 |
| Total | 1,217,419 | 1,174,767 | 42652 |  | 268,872,787 |  |

Supplemental table S3: Numbers and characters of sequence reads in 2017.

| Sample name | Total reads | Combined  reads | Uncombined  reads | Combined  Percentage (%) | Combined  base(bp) | Average length (bp) |
| --- | --- | --- | --- | --- | --- | --- |
| bulksoil4 | 70,017 | 67,227 | 2,790 | 96.02 | 14,441,003 | 215 |
| bulksoil5 | 61,857 | 59,231 | 2,626 | 95.75 | 12,588,711 | 213 |
| bulksoil6 | 79,393 | 75,598 | 3,795 | 95.22 | 15,466,082 | 205 |
| N_LN1_1 | 61,203 | 59,360 | 1,843 | 96.99 | 13,993,366 | 236 |
| N_LN1_2 | 63,962 | 61,267 | 2,695 | 95.79 | 13,127,158 | 214 |
| R_LN1_1 | 95,503 | 91,943 | 3,560 | 96.27 | 19,416,411 | 211 |
| R_LN1_2 | 57,470 | 55,080 | 2,390 | 95.84 | 11,609,607 | 211 |
| N_LN2_1 | 54,475 | 52,808 | 1,667 | 96.94 | 11,877,547 | 225 |
| N_LN2_2 | 72,494 | 70,789 | 1,705 | 97.65 | 14,603,854 | 206 |
| R_LN2_1 | 55,546 | 52,885 | 2,661 | 95.21 | 12,029,856 | 227 |
| R_LN2_2 | 53,614 | 51,642 | 1,972 | 96.32 | 11,217,469 | 217 |
| N_LN3_1 | 73,230 | 70,857 | 2,373 | 96.76 | 15,631,804 | 221 |
| N_LN3_2 | 70,607 | 67,187 | 3,420 | 95.16 | 14,447,600 | 215 |
| R_LN3_1 | 71,381 | 69,153 | 2,228 | 96.88 | 15,989,404 | 231 |
| R_LN3_2 | 65,524 | 61,619 | 3,905 | 94.04 | 13,536,469 | 220 |
| N_LN4_1 | 58,965 | 53,834 | 5,131 | 91.3 | 11,527,038 | 214 |
| N_LN4_2 | 72,467 | 69,593 | 2,874 | 96.03 | 15,245,474 | 219 |
| R_LN4_1 | 58,772 | 56,105 | 2,667 | 95.46 | 11,947,917 | 213 |
| R_LN4_2 | 82,853 | 78,543 | 4,310 | 94.8 | 16,288,877 | 207 |
| N_HN1_1 | 80,527 | 77,753 | 2,774 | 96.56 | 17,320,516 | 223 |
| N_HN1_2 | 64,929 | 62,290 | 2,639 | 95.94 | 13,596,496 | 218 |
| R_HN1_1 | 71,121 | 68,827 | 2,294 | 96.77 | 15,374,296 | 223 |
| R_HN1_2 | 71,281 | 69,552 | 1,729 | 97.57 | 14,995,617 | 216 |
| N_HN2_1 | 66,536 | 63,440 | 3,096 | 95.35 | 13,911,843 | 219 |
| N_HN2_2 | 62,443 | 59,540 | 2,903 | 95.35 | 13,979,876 | 235 |
| R_HN2_1 | 71,278 | 68,372 | 2,906 | 95.92 | 13,301,483 | 195 |
| R_HN2_2 | 86,919 | 72,403 | 14,516 | 83.3 | 14,680,200 | 203 |
| N_HN3_1 | 71,496 | 68,520 | 2,976 | 95.84 | 15,562,926 | 227 |
| N_HN3_2 | 88,007 | 84,146 | 3,861 | 95.61 | 19,143,861 | 228 |
| R_HN3_1 | 72,888 | 70,900 | 1,988 | 97.27 | 16,265,886 | 229 |
| R_HN3_2 | 70,081 | 65,057 | 5,024 | 92.83 | 14,007,110 | 215 |
| N_HN4_1 | 68,837 | 65,389 | 3,448 | 94.99 | 15,075,648 | 231 |
| N_HN4_2 | 73,816 | 71,626 | 2,190 | 97.03 | 16,752,164 | 234 |
| R_HN4_1 | 76,884 | 73,928 | 2,956 | 96.16 | 17,652,630 | 239 |
| R_HN4_2 | 80,657 | 78,166 | 2,491 | 96.91 | 17,699,449 | 226 |
| Total | 2,457,033 | 2,344,630 | 112,403 |  | 514,305,648 |  |

Supplemental table S4: Results of read quality control in 2016.

| Sample Name | Raw PE(#) | Combined(#) | Qualified(#) | Nochime(#) | AvgLen(nt) | Q20 | Q30 | GC% | Effective% |
| --- | --- | --- | --- | --- | --- | --- | --- | --- | --- |
| Bulksoil1 | 89,486 | 85,898 | 85,159 | 83,741 | 217 | 99.11 | 98.37 | 43.68 | 93.58 |
| Bulksoil2 | 89,282 | 85,634 | 84,633 | 83,243 | 218 | 99.05 | 98.26 | 42.92 | 93.24 |
| Bulksoil3 | 87,147 | 84,159 | 82,951 | 81,526 | 234 | 99.01 | 98.07 | 47.72 | 93.55 |
| N_P2_1 | 80,675 | 78,556 | 76,386 | 75,575 | 238 | 98.99 | 98 | 50.73 | 93.68 |
| N_P2_2 | 73,690 | 71,683 | 70,700 | 69,661 | 223 | 99.14 | 98.33 | 48.08 | 94.53 |
| N_P2_3 | 74,647 | 72,094 | 71,114 | 70,012 | 226 | 98.97 | 98.02 | 45.85 | 93.79 |
| N_P1_1 | 85,407 | 82,336 | 81,165 | 79,882 | 232 | 98.98 | 98.04 | 46.53 | 93.53 |
| N_P1_2 | 83,038 | 80,013 | 78,842 | 77,501 | 228 | 99.09 | 98.23 | 47.51 | 93.33 |
| N_P1_3 | 70,284 | 66,806 | 62,727 | 61,098 | 225 | 99.05 | 98.22 | 43.67 | 86.93 |
| R_P2_1 | 73,615 | 71,652 | 70,634 | 69,777 | 237 | 99.04 | 98.08 | 51.03 | 94.79 |
| R_P2_2 | 77,693 | 74,953 | 73,946 | 72,602 | 222 | 99.11 | 98.29 | 47.28 | 93.45 |
| R_P2_3 | 89,257 | 85,113 | 82,870 | 81,671 | 229 | 99.02 | 97.97 | 49.05 | 91.5 |
| R_P1_1 | 81,844 | 79,558 | 78,455 | 77,131 | 231 | 99.02 | 98.07 | 47.23 | 94.24 |
| R_P1_2 | 71,910 | 69,912 | 67,644 | 66,734 | 234 | 99.05 | 98.11 | 49.63 | 92.8 |
| R_P1_3 | 89,444 | 86,400 | 85,234 | 83,589 | 226 | 99.02 | 98.13 | 46.17 | 93.45 |

Supplemental table S5: Results of read quality control in 2017.

| Sample Name | Raw PE(#) | Combined(#) | Qualified(#) | Nochime(#) | AvgLen(nt) | Q20 | Q30 | GC% | Effective% |
| --- | --- | --- | --- | --- | --- | --- | --- | --- | --- |
| N_HN3_1 | 71,496 | 68,520 | 64,867 | 63,405 | 229 | 99.05 | 98.2 | 46.88 | 88.68 |
| N_HN3_2 | 88,007 | 84,146 | 82,854 | 81,367 | 226 | 99.05 | 98.15 | 47.44 | 92.46 |
| N_LN2_1 | 54,475 | 52,808 | 52,044 | 50,998 | 224 | 99.09 | 98.27 | 46.78 | 93.62 |
| N_LN2_2 | 72,494 | 70,789 | 70,169 | 67,558 | 206 | 99.5 | 98.98 | 43.72 | 93.19 |
| N_HN4_1 | 68,837 | 65,389 | 63,265 | 61,435 | 231 | 99.05 | 98.14 | 42.21 | 89.25 |
| N_HN4_2 | 73,816 | 71,626 | 70,176 | 68,552 | 233 | 98.74 | 97.71 | 50.18 | 92.87 |
| N_HN1_1 | 80,527 | 77,753 | 76,999 | 75,471 | 221 | 99.06 | 98.26 | 43.86 | 93.72 |
| N_HN1_2 | 64,929 | 62,290 | 60,038 | 58,751 | 220 | 99.21 | 98.52 | 44.67 | 90.48 |
| N_LN3_1 | 73,230 | 70,857 | 69,970 | 68,048 | 219 | 99.11 | 98.34 | 43.44 | 92.92 |
| N_LN3_2 | 70,607 | 67,187 | 64,740 | 62,948 | 215 | 99.2 | 98.55 | 40.82 | 89.15 |
| N_HN2_1 | 66,536 | 63,440 | 60,783 | 59,225 | 220 | 99.01 | 98.09 | 43.07 | 89.01 |
| N_HN2_2 | 62,443 | 59,540 | 57,260 | 56,031 | 235 | 98.78 | 97.59 | 41.47 | 89.73 |
| N_LN4_1 | 58,965 | 53,834 | 53,088 | 51,616 | 213 | 99.11 | 98.36 | 42.72 | 87.54 |
| N_LN4_2 | 72,467 | 69,593 | 68,821 | 67,358 | 218 | 99.16 | 98.4 | 44.68 | 92.95 |
| Bulksoil4 | 70,017 | 67,227 | 66,448 | 65,053 | 213 | 99.15 | 98.43 | 43.24 | 92.91 |
| Bulksoil5 | 61,857 | 59,231 | 58,497 | 56,877 | 211 | 99.2 | 98.56 | 43.08 | 91.95 |
| Bulksoil6 | 79,393 | 75,598 | 74,970 | 73,368 | 203 | 99.19 | 98.59 | 39.76 | 92.41 |
| R_LN1_1 | 95,503 | 91,943 | 91,193 | 88,680 | 211 | 99.1 | 98.36 | 48.09 | 92.86 |
| R_LN1_2 | 57,470 | 55,080 | 54,456 | 53,152 | 210 | 99.14 | 98.43 | 42.24 | 92.49 |
| R_HN3_1 | 72,888 | 70,900 | 69,871 | 68,752 | 229 | 99.15 | 98.33 | 47.49 | 94.33 |
| R_HN3_2 | 70,081 | 65,057 | 60,732 | 58,786 | 217 | 99.1 | 98.37 | 40.64 | 83.88 |
| R_LN2_1 | 55,546 | 52,885 | 50,986 | 49,615 | 228 | 98.95 | 97.94 | 41.91 | 89.32 |
| R_LN2_2 | 53,614 | 51,642 | 49,934 | 48,708 | 217 | 99.16 | 98.48 | 41.81 | 90.85 |
| R_HN4_1 | 76,884 | 73,928 | 71,370 | 70,119 | 239 | 98.98 | 97.98 | 48.35 | 91.2 |
| R_HN4_2 | 80,657 | 78,166 | 76,645 | 75,365 | 226 | 99.13 | 98.35 | 42.74 | 93.44 |
| R_HN1_1 | 71,121 | 68,827 | 68,124 | 66,819 | 222 | 99.13 | 98.37 | 43.38 | 93.95 |
| R_HN1_2 | 71,281 | 69,552 | 68,985 | 67,595 | 215 | 99.24 | 98.61 | 40.99 | 94.83 |
| R_LN3_1 | 71,381 | 69,153 | 68,022 | 66,782 | 231 | 99.13 | 98.31 | 46.44 | 93.56 |
| R_LN3_2 | 65,524 | 61,619 | 60,439 | 59,112 | 219 | 99.12 | 98.32 | 43.92 | 90.21 |
| R_HN2_1 | 71,278 | 68,372 | 68,044 | 66,119 | 193 | 99.53 | 99.03 | 40.87 | 92.76 |
| R_HN2_2 | 86,919 | 72,403 | 71,922 | 70,031 | 202 | 99.48 | 98.94 | 42.43 | 80.57 |
| R_LN4_1 | 58,772 | 56,105 | 55,535 | 54,226 | 212 | 99.18 | 98.49 | 43.95 | 92.27 |
| R_LN4_2 | 82,853 | 78,543 | 77,806 | 76,106 | 206 | 99.18 | 98.5 | 43.59 | 91.86 |

Supplemental table S6: Results of tag annotation and OTU cluster analysis in 2016.

| Sample_Name | Total_tag | Taxon_Tag | Unclassified_Tag | Unique_Tag | OTU_num |
| --- | --- | --- | --- | --- | --- |
| Bulksoil1 | 83741 | 57188 | 25640 | 913 | 1119 |
| Bulksoil2 | 83243 | 67389 | 15089 | 765 | 1184 |
| Bulksoil3 | 81526 | 34022 | 46511 | 993 | 1164 |
| N_P2_1 | 75575 | 29298 | 45595 | 682 | 1125 |
| N_P2_2 | 69661 | 38438 | 30365 | 858 | 1220 |
| N_P2_3 | 70012 | 36202 | 32769 | 1041 | 1281 |
| N_P1_1 | 79882 | 38008 | 40837 | 1037 | 1278 |
| N_P1_2 | 77501 | 40150 | 36206 | 1145 | 1308 |
| N_P1_3 | 61098 | 39015 | 20996 | 1087 | 1209 |
| R_P2_1 | 69777 | 22175 | 46787 | 815 | 1073 |
| R_P2_2 | 72602 | 44544 | 27058 | 1000 | 1286 |
| R_P2_3 | 81671 | 42550 | 38172 | 949 | 1158 |
| R_P1_1 | 77131 | 32813 | 43197 | 1121 | 1154 |
| R_P1_2 | 66734 | 32128 | 33975 | 631 | 1032 |
| R_P1_3 | 83589 | 51397 | 30960 | 1232 | 1295 |

Supplemental table S7: Results of tag annotation and OTU cluster analysis in 2017.

| Sample_Name | Total_tag | Taxon_Tag | Unclassified_Tag | Unique_Tag | OTU_num |
| --- | --- | --- | --- | --- | --- |
| N_LN1_1 | 57400 | 23330 | 33298 | 772 | 1021 |
| N_LN1_2 | 59122 | 46882 | 11244 | 996 | 1158 |
| N_HN3_1 | 63405 | 38510 | 23979 | 916 | 1108 |
| N_HN3_2 | 81367 | 32830 | 47277 | 1260 | 1374 |
| N_LN2_1 | 50998 | 33538 | 16900 | 560 | 1005 |
| N_LN2_2 | 67558 | 59483 | 7694 | 381 | 589 |
| N_HN4_1 | 61435 | 50098 | 10660 | 677 | 1023 |
| N_HN4_2 | 68552 | 48594 | 19281 | 677 | 994 |
| N_HN1_1 | 75471 | 49995 | 24372 | 1104 | 1374 |
| N_HN1_2 | 58751 | 44543 | 13502 | 706 | 977 |
| N_LN3_1 | 68048 | 54894 | 12311 | 843 | 1264 |
| N_LN3_1 | 62948 | 46018 | 15989 | 941 | 1159 |
| N_HN2_1 | 59225 | 48018 | 10424 | 783 | 1034 |
| N_HN2_2 | 56031 | 47319 | 8053 | 659 | 868 |
| N_LN4_1 | 51616 | 32197 | 17880 | 1539 | 1317 |
| N_LN4_2 | 67358 | 47925 | 18238 | 1195 | 1367 |
| Bulksoil4 | 65053 | 53688 | 10607 | 758 | 1228 |
| Bulksoil5 | 56877 | 47516 | 8549 | 812 | 1191 |
| Bulksoil6 | 73368 | 33190 | 38848 | 1330 | 1288 |
| R_LN1_1 | 88680 | 78774 | 8705 | 1201 | 1215 |
| R_LN1_2 | 53152 | 30388 | 21930 | 834 | 1106 |
| R_HN3_1 | 68752 | 37920 | 29904 | 928 | 1096 |
| R_HN3_2 | 58786 | 35005 | 22341 | 1440 | 1207 |
| R_LN2_1 | 49615 | 37992 | 10849 | 774 | 992 |
| R_LN2_2 | 48708 | 28677 | 19208 | 823 | 893 |
| R_HN4_1 | 70119 | 33404 | 36044 | 671 | 1018 |
| R_HN4_2 | 75365 | 58999 | 15574 | 792 | 1154 |
| R_HN1_1 | 66819 | 44544 | 21220 | 1055 | 1170 |
| R_HN1_2 | 67595 | 37918 | 28737 | 940 | 1026 |
| R_LN3_1 | 66782 | 30966 | 35171 | 645 | 961 |
| R_LN3_2 | 59112 | 26549 | 31688 | 875 | 919 |
| R_HN2_1 | 66119 | 55534 | 9747 | 838 | 722 |
| R_HN2_2 | 70031 | 62668 | 6785 | 578 | 730 |
| R_LN4_1 | 54226 | 41083 | 12266 | 877 | 1255 |
| R_LN4_2 | 76106 | 54119 | 20534 | 1453 | 1513 |

Supplemental table S8: The character of QTLs and nodulation of soybean plants

| Line^a^ | *qBNF-C2^b^* | |  | *qBNF-O* | |  | *qBNF-B1* | | Nodule number |
| --- | --- | --- | --- | --- | --- | --- | --- | --- | --- |
|  | Satt286 | Satt281 |  | Satt592 | Satt190 |  | Satt509 | Satt272 |  |
| P1 | - | - |  | + | + |  | - | - | 21.5 ± 6.9 |
| P2 | + | + |  | - | - |  | - | - | 76 ± 24.9 |
| HN1 | + | + |  | - | - |  | + | + | 102 ± 19 |
| HN2 | + | + |  | + | + |  | + | + | 150 ± 20.3 |
| HN3 | - | - |  | + | + |  | + | + | 105 ± 13.6 |
| HN4 | + | + |  | + | + |  | - | - | 90.8 ± 11.7 |
| LN1 | - | - |  | - | - |  | - | - | 19.8 ± 9.0 |
| LN2 | - | - |  | - | - |  | - | - | 24.3 ± 7.9 |
| LN3 | - | - |  | - | - |  | - | - | 20 ± 7 |
| LN4 | - | - |  | - | - |  | - | - | 24.1 ± 7.8 |

Note: ^a^ P1 and P2 are parental lines, while HN1-4 and LN1-4 are selected from RIL population by six flanking markers which were identified in previously study (Yang et al., 2017). ^b^ “-” and “+” mean the negative and positive effective alleles derived from parents, respectively.
